# An Integrated in Silico, in Vitro, and in Vivo Assessment of the Antioxidant and Neuroprotective Profile of Selenocyanate Compounds

**DOI:** 10.1007/s12011-026-05247-7

**Published:** 2026-07-31

**Authors:** Maria Eduarda Ribeiro da Rocha, Bruna Rafaela Pizzi, Pâmella da Costa, Tácia Katiane Hall, Mariana Parron Paim, Pâmella Cordeiro, Victor Menezes, Vanessa Nascimento, César Augusto Brüning, Paloma Taborda Birmann, Cristiani Folharini Bortolatto

**Affiliations:** 1 Laboratory of Biochemistry and Molecular Neuropharmacology (LABIONEM), Graduate Program in Biochemistry and Bioprospecting (PPGBBio), Chemical, Pharmaceutical, and Food +Sciences Center (CCQFA), Federal University of Pelotas (UFPel), Capão do Leão Campus, Pelotas, RS 96010-900 Brazil; 2 SupraSelen Laboratory, Chemistry Institute, Department of Organic Chemistry, Federal Fluminense University (UFF), Niterói, Rio de Janeiro, Brazil; 3 Research Group in Biochemistry and Toxicology in Eukaryotes, Postgraduate Program in Biochemistry, Federal University of Pampa (UNIPAMPA), Bagé, RS Brazil

**Keywords:** Selenium, Cyanate, Oxidative stress, Monoamine oxidase, Acetylcholinesterase, Toxicity

## Abstract

Cyanate-based compounds have received increasing attention due to their neuroprotective and antioxidant properties, which can be further enhanced by the incorporation of selenium, a key element in redox regulation. In this study, five selenocyanate derivatives (**1**–**5**), differing by alkyl or aryl groups, were synthesized and systematically evaluated using an integrated in silico, in vitro, and in vivo approach to explore their pharmacological and toxicological potential. Computational analyses included predictions of pharmacokinetic and toxicological parameters (ADME/T), as well as molecular docking studies targeting neurotransmitter-related enzymes, namely acetylcholinesterase (AChE) and monoamine oxidases (MAO-A and B). Antioxidant activity was evaluated using multiple complementary in vitro assays, including DPPH, ABTS, FRAP, GST-like, and SOD-like, as well as cerebral reactive species (RS). Cerebral AChE, MAO-A, and MAO-B activities were also measured in vitro. In vivo experiments involved evaluating acute oral toxicity in female Swiss mice. In silico results indicated favorable ADME/T profiles and affinity of selenocyanates for the target neurochemical enzymes. In vitro assays revealed antioxidant capacity for selenocyanates, particularly for compounds **1**, **4**, and **5**. All compounds inhibited cerebral AChE and MAO isoforms in vitro, with particular emphasis on compounds **2** and **4** as AChE inhibitors. Therefore, compounds **2** and **4 **were tested in vivo and showed no acute toxicity at an oral dose of 50 mg/kg; however, at 300 mg/kg, compound **2** showed signs of toxicity, whereas compound **4** remained non-lethal. Taken together, these results highlight organoselenocyanates, especially compound** 4**, as promising candidates for the development of antioxidant and neuroprotective agents.

**Graphical Abstract:**

**Figure:**
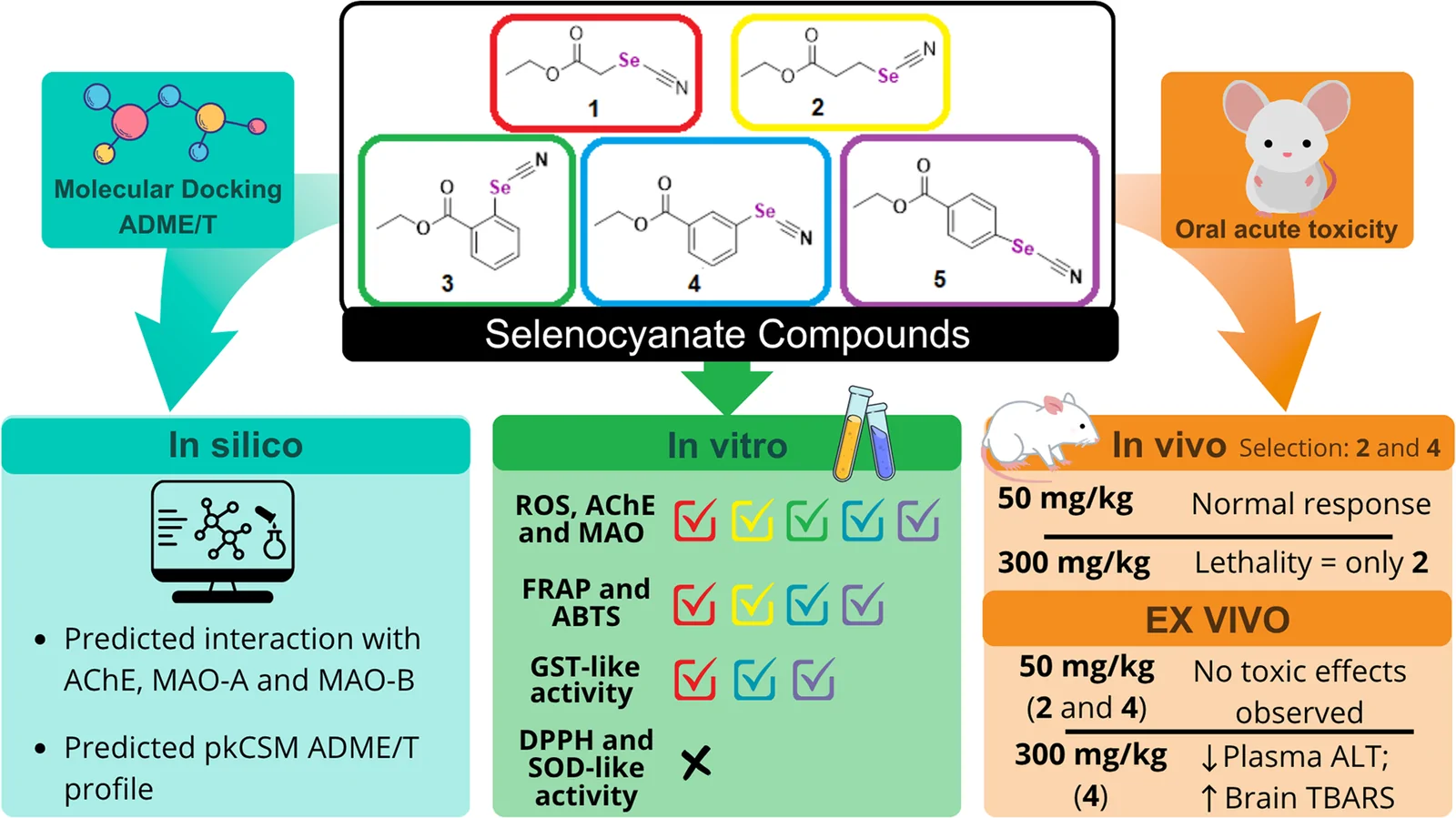

**Supplementary Information:**

The online version contains supplementary material available at https://doi.org/10.1007/s12011-026-05247-7.

## Introduction

Organic selenocyanates (RSeCN) constitute a distinct and promising class of organoselenium compounds, characterized by the presence of selenium covalently bound to carbon frameworks and a cyanate moiety. Owing to their synthetic versatility and chemical stability, these compounds have attracted increasing attention in medicinal chemistry, particularly due to their biological activities [1]. Experimental evidence has demonstrated that some selenocyanates exhibit relevant antioxidant and detoxifying properties. For instance, diphenylmethyl selenocyanate showed a chemoprotective effect against malachite green–induced hepatotoxicity, highlighting its potential as an effective antioxidant agent [2].

The biological relevance of selenocyanates may be attributed to the essential role of selenium as a trace element in human physiology. Selenium acts as a crucial cofactor for several antioxidant enzymes, including glutathione peroxidase (GPx), thioredoxin reductase, and other selenoproteins, which are fundamental for maintaining redox homeostasis [3]. The incorporation of selenium atoms into organic molecules has been shown to significantly enhance their biological activities, such as antioxidant, antibacterial, antitumor, and chemopreventive effects [4, 5].

A notable example is ebselen, a well-established organoselenium compound with glutathione peroxidase-like activity, which has been extensively investigated for the treatment of various human diseases [6]. In addition, several organoselenium compounds [7–9], including selenocyanate derivatives conjugated with non-steroidal anti-inflammatory drugs [10], have been reported to exert neuroprotective effects. Together, these reports underscore the relevance of selenocyanates as a valuable subclass of organoselenium compounds and as promising candidates for the development of novel antioxidants and neuroprotective agents.

The relevance of such compounds becomes particularly evident in chronic diseases in which persistent oxidative stress (OS) is a central pathogenic mechanism, including neurodegenerative disorders and psychiatric disorders. Increasing evidence indicates that neuronal injury in neurodegenerative diseases arises from the convergence of multiple interconnected processes, including impaired energy metabolism, dysregulated calcium homeostasis, abnormal mitochondrial dynamics, and OS [11].

Alzheimer’s disease the oxidative damage and mitochondrial dysfunction are significantly exacerbated compared to normal aging and are already evident during the prodromal stages of the disease, contributing to early neuronal impairment [12]. Growing evidence also implicates OS in the pathophysiology of several psychiatric conditions, such as major depressive disorder, where redox imbalance, mitochondrial dysfunction, and neuroinflammation have been associated with the onset, progression, and severity of disease symptoms [13]. Taken together, these findings highlight OS as a common and critical pathological denominator in both neurodegenerative and psychiatric disorders.

Clinically, monoamine oxidase A (MAO-A), monoamine oxidase B (MAO-B), and AChE inhibitors are widely used as antidepressants, antiparkinsonian agents, and anti-Alzheimer’s drugs, respectively [14, 15]. Despite the recognized redox-modulating properties of organoselenium compounds, the potential of selenocyanate derivatives as multitarget agents combining antioxidant activity with MAO and AChE inhibition remains largely unexplored.

Therefore, the present study aimed to synthesize five selenocyanate derivatives and evaluate their in vitro antioxidant activity, as well as their in vitro and in silico inhibitory potential against MAO and AChE. Furthermore, the toxicological profiles of all derivatives were predicted through in silico ADME/T analyses, and two selected compounds were further investigated for acute oral toxicity in female Swiss mice, generating in vivo and ex vivo evidence regarding their safety profile. Supplementary predicted physicochemical parameters were also generated to better complement and support the toxicological data.

## Materials and Methods

The study comprised four sequential stages: (i) synthesis and structural characterization of the compounds; (ii) in silico analyses, including ADMET profiling and molecular docking against MAO and AChE; (iii) in vitro evaluation of antioxidant activity and inhibitory effects on cerebral MAO and AChE; and (iv) an acute oral toxicity assessment, providing both in vivo and ex vivo evidence of biological safety of the selected compounds, along with supplementary physicochemical parameters, generated to support the toxicological data.

### Chemicals

**Fig. 1:**
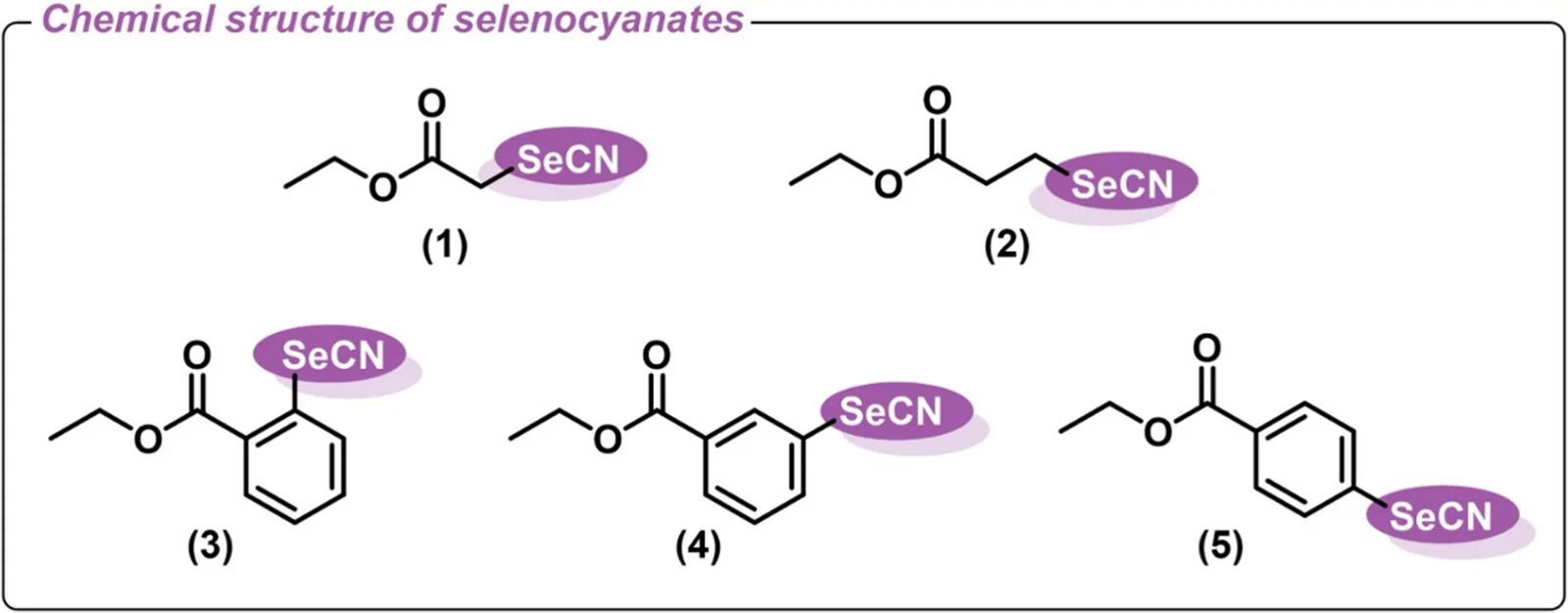
Chemical structures of selenocyanates 1–5. Ethyl acetate 2-selenocyanate **(1)**, ethyl propanoate 3-selenocyanate **(2)**, ethyl benzoate 2-selenocyanate **(3)**, ethyl benzoate 3-selenocyanate **(4)**, and ethyl benzoate 4-selenocyanate **(5)**

The SupraSelen Laboratory at the Federal Fluminense University (UFF, Brazil) synthesized a series of selenocyanate compounds (**1–5**) with unknown antioxidant properties. These molecules consist of a selenium atom bonded to a cyanate group and are arranged in ester chains, namely: ethyl acetate 2-selenocyanate **(1)**, ethyl propanoate 3-selenocyanate **(2)**, ethyl benzoate 2-selenocyanate **(3)**, ethyl benzoate 3-selenocyanate **(4)**, and ethyl benzoate 4-selenocyanate **(5)**. The corresponding chemical structures are shown in Fig. 1.

**Fig. 2:**
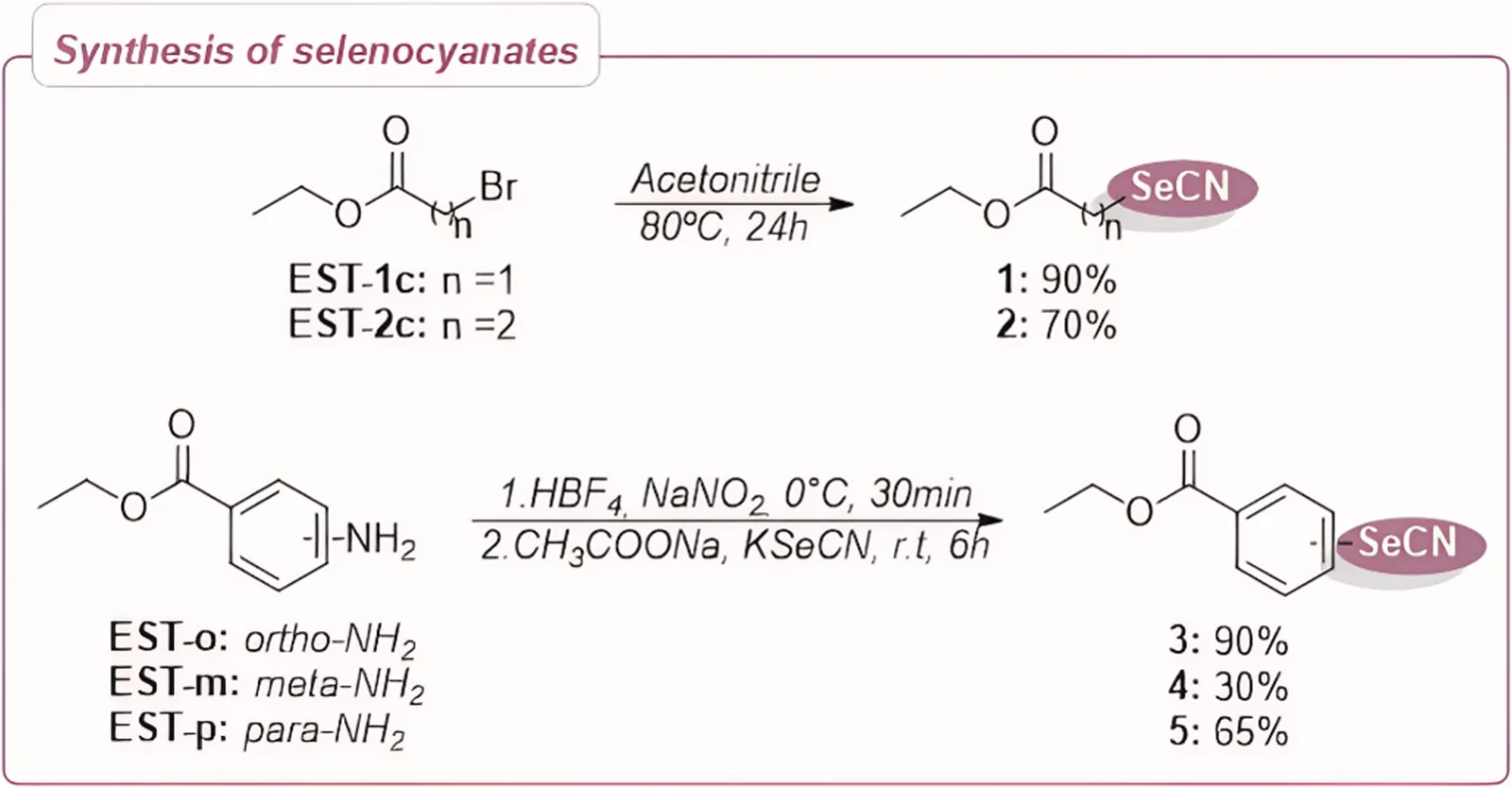
Synthesis of the selenocyanates **1–5**

The synthesis of the ethyl 2-selenocyanatoacetate **(1)** and ethyl 3-selenocyanatopropanoate **(2)** was performed following the protocol established in the previous study (Fig. 2). Respective bromide **EST-1c** or **EST-2c** and underwent nucleophilic substitution with potassium selenocyanate in acetonitrile under reflux for 24 h, furnishing the corresponding products in 90% and 70% yield, respectively [16]. The ethyl 2-selenocyanatobenzoate **(3)**, ethyl 3-selenocyanatobenzoate **(4)** and ethyl 4-selenocyanatobenzoate **(5)** were synthesized following a previously reported literature procedure (Fig. 2). In this method, the corresponding aryl amines **EST(o-p)** were subjected to a diazotization-selenocyanation sequence, in which the in situ-generated diazonium salts reacted with potassium selenocyanate to afford the target compounds as yellow oils in 30–90% yield [17]. Chemical synthesis can be found in the supplementary materials (S1–S10).

Due to their lipophilic nature, selenocyanate compounds were solubilized in dimethyl sulfoxide (DMSO) for in vitro assays or in canola oil for toxicological experiments. Ascorbic acid (AA), 6-hydroxy-2,5,7,8-tetramethylchromano-2-carboxylic acid (Trolox), butylated hydroxytoluene (BHT), rivastigmine, clorgiline, and pargyline, all obtained from Merck, were used as positive controls in in vitro assays, along with the organoselenium compound diphenyl diselenide (PhSe)₂, which was synthesized in collaboration with a partner laboratory (SustenSin).

### Animals

Sixty-day-old male and female Swiss mice weighing approximately 25–35 g were obtained from the Central Animal Facility of the Federal University of Pelotas (UFPel). The animals were kept at a controlled temperature (22 ± 1 °C), 12-h light/dark cycle, relative humidity (60% ± 20%) and free access to water and standard chow. The animals were housed in groups of 3 per polycarbonate box, measuring 30 × 20 × 13 cm. The experiments were approved by the UFPel Animal Use Ethics Committee (CEUA) under number 014520/2025-14, affiliated with the National Council for Animal Experimentation Control (CONCEA). Animals were euthanized by isoflurane overdose, followed by cervical dislocation. Isoflurane was also used to anesthetize the animals prior to blood collection by cardiac puncture. Biological samples (brain and blood plasma) were stored individually in microtubes at − 80 °C until analysis. For in vitro, whole brain tissue from the male and female mice was used for the following assays: MAO activity (isoforms A and B), AChE activity, and determination of RS levels, in which a pool of tissues was prepared for each technique. The use of mixed-sex brain tissue in vitro was intended to capture an overall basal neurochemical response at the initial screening stage, rather than to resolve sex-specific differences.

### In Silico Assays

In silico techniques refer to the use of computational tools that allow the prediction of biological, chemical, or pharmacological phenomena without the need for direct laboratory experimentation. In this study, in silico analyses were initially performed to evaluate absorption, distribution, metabolism, excretion, and toxicity (ADME/T) properties, as well as to predict binding affinity and mode of interaction with the target proteins (MAO-A, MAO-B, and AChE enzymes) through molecular docking.

#### Prediction of ADME/T Properties and Physicochemical Properties

ADME/T predictions for the compounds **1**–**5** were performed using the pkCSM ADME/T algorithm (Accelrys Software, Inc., San Diego, CA, USA) [18]. The toxicity profile of the selenocyanate compounds was assessed through a comprehensive set of in silico parameters, including mutagenic potential (AMES test), intestinal absorption, hepatotoxicity, and blood–brain barrier (BBB) permeability. Excretion properties were predicted using a total clearance model. Metabolic liability was estimated based on cytochrome P450 (CYP) models, determining whether the compounds act as inhibitors or substrates of the major CYP isoforms. Distribution characteristics were analyzed considering BBB permeability and central nervous system (CNS) penetration. Absorption was evaluated through Caco-2 cell permeability and intestinal absorption. Toxicity was predicted through AMES toxicity, oral rat acute toxicity (LD_50_), oral rat chronic toxicity (LOAEL) and hepatotoxicity.

Physicochemical properties of the two most promising in vitro compounds were predicted using ADMETlab 3.0 (Xiangya School of Pharmaceutical Sciences, Central South University) [19], including lipophilicity (logP and logD), pKa, and human liver microsomal (HLM) stability, as relevant parameters for structure–activity and biological activity comparisons.

#### Molecular Docking Analysis

Molecular docking was employed to predict the binding energies, optimal orientations, and binding conformations of ligands (compounds **1–5**) with the MAO-A, MAO-B, and AChE isoforms. This technique enabled the simulation and analysis of interactions between the tested compounds and the target proteins. The following drugs were used as positive controls: clorgyline (a selective MAO-A inhibitor), pargyline (a selective MAO-B inhibitor), rivastigmine (an AChE inhibitor).

The two-dimensional (2D) structures of the ligands were initially drawn using ChemDraw and subsequently converted into three-dimensional (3D) format using Avogadro 0.9.4 [20]. AutoDock Tools MGLTools package (ADT) (version 1.5.7) [21] was used to assign atomic charges, as well as define torsional flexibility for each molecule. The target proteins were obtained in the RCSB Protein Data Bank, their IDs were retrieved from the literature human MAO-A (PDB ID: 2Z5X) [22], human MAO-B (PDB ID: 1GOS) [23], and human AChE (PDB ID: 7E3H) [24]. Native ligands were removed from the protein structures using PyMOL 3.0.0 [25]. Enzyme preparation, which included the removal of water molecules, ions, and heteroatoms, and the grid box definition was performed using AutoDock Tools. Docking simulations were carried out using AutoDock Vina 1.1.1 [26], which searches for the most favorable binding conformations. Finally, ligand–protein interactions were visualized and analyzed using Discovery Studio 2016 version 24.1.0.23298 [27].

### In Vitro Assays

The following subsections describe the methodology employed in the in vitro assays conducted to investigate the antioxidant activity and enzyme inhibition potential of selenocyanate compounds. RS levels, MAO, and AChE activities were measured in mouse brain tissue while the ferric ion reducing power (FRAP), 2,2′-azino-bis(3-ethylbenzothiazoline-6-sulfonic acid) (ABTS), 2,2-diphenyl-1-picrylhydrazyl (DPPH), Glutathione S-transferase enzyme mimetic (GST-like) and superoxide dismutase enzyme mimetic (SOD-like) assays were performed in cell-/tissue-free systems. All analyses were performed with three independent replicates (*n* = 3), except for the RS analysis (*n* = 4). The control group for the in vitro techniques received reaction reagents plus vehicle (DMSO) used to solubilize the compounds, at the same volume (10 µL) as that used in the compound groups, thereby controlling for any potential effects of the solvent itself.

The compounds were evaluated at two concentrations (10 and 200 µM), both within a range widely employed in studies with organoselenium compounds. Preliminary screening also revealed a strong inhibitory effect on AChE activity, prompting additional assays at lower concentrations (5–20 µM) to better characterize the potency of enzyme inhibition.

#### FRAP Assay

The FRAP was performed in an acellular system to evaluate antioxidant capacity based on the reduction of Fe³⁺ to Fe²⁺ under acidic conditions, following a protocol adapted from Yoshino and Murakami [28]. This method is based on the electron donation capacity of antioxidant compounds, which reduce the ferric tripyridyltriazine complex (Fe³⁺–TPTZ), characterized by a yellow color, to the ferrous form (Fe²⁺–TPTZ), producing an intense blue complex. The increase in color intensity is directly proportional to the antioxidant capacity of the compounds tested [29].

The FRAP solution was freshly prepared by mixing 38 mM sodium acetate buffer (pH 3.6), 20 mM ferric chloride (FeCl₃), and 10 mM 2,4,6-tris(2-pyridyl)-s-triazine (TPTZ) dissolved in 40 mM HCl in a 10:1:1 (v/v/v) ratio. The reaction mixture was incubated for 30 min with the carrier (DMSO, control), selenocyanates (**1–5**) at final concentrations of 10 or 200 µM, or the positive control (AA, Cf: 10 µM). After incubation, the formation of the Fe²⁺–TPTZ complex was quantified spectrophotometrically by measuring the absorbance at 593 nm. The results were expressed as absorbance values.

#### ABTS Radical Scavenging Activity Assay

Adapted from Re et al. [30], this cell-free assay evaluates antioxidant activity based on the scavenging of the free radical 2,2′-azino-bis(3-ethylbenzothiazoline-6-sulfonic acid) (ABTS•⁺) by antioxidant compounds. The ABTS•⁺ radical is generated through oxidation of ABTS by potassium persulfate, promoting the conversion of the reduced, colorless form into its oxidized blue–green chromophore. The ABTS•⁺ solution (Cf: 42.77 µM) was incubated for 30 min with vehicle (DMSO) as control, selenocyanates **1–5** at final concentrations of 10 or 200 µM, or the positive control (AA, Cf: 25 µM). After incubation, absorbance was measured spectrophotometrically at 734 nm. Antioxidant activity was expressed as the percentage of ABTS•⁺ radical scavenging, calculated relative to the blank (tube containing the reaction components but without the DMSO vehicle), which was defined as 100%.

#### DPPH Radical Scavenger Activity Assay

According to the protocol described by Sharma and Bhat [31], method is based on the ability of antioxidants to neutralize the stable free radical 2,2-diphenyl-1-picrylhydrazyl (DPPH•), primarily through hydrogen atom donation. Reduction of DPPH• radical results in a characteristic color change from violet to yellow, which can be quantitatively monitored. A DPPH solution (Cf: 50 µM) was incubated for 30 min with vehicle (DMSO) as control, selenocyanates **1–5** at final concentrations of 10 or 200 µM, or the positive control (AA, Cf: 25 µM). After incubation, absorbance was measured spectrophotometrically at 517 nm. Antioxidant activity was expressed as the percentage of DPPH• radical reduction, with the blank sample considered as 100%.

#### GST-like Activity Test

This cell-free assay evaluated the ability of the tested compounds to mimic GST, a key enzyme involved in antioxidant defense and cellular detoxification. Compounds exhibiting GST-like activity can facilitate the conjugation of reduced glutathione (GSH) with electrophilic substrates, thereby promoting the neutralization of RS. This activity was assessed through the conjugation reaction between GSH and 1-chloro-2,4-dinitrobenzene (CDNB), following a protocol adapted from Habig et al. [32].

The experimental groups comprised a control group incubated for 2 min with vehicle (DMSO), groups incubated with selenocyanates **1**–**5** at final concentrations of 10 or 200 µM, and a positive control diphenyl diselenide [(PhSe)₂, Cf: 25 µM]. The substrates GSH (Cf: 2 mM) and CDNB (Cf: 1 mM) were added in a sodium phosphate buffer medium. The formation of the GSH–CDNB conjugate was monitored spectrophotometrically at 340 nm over a 3 min period. GST-like activity was expressed as the change in absorbance per minute, reflecting the catalytic efficiency of the tested compounds in GSH conjugation.

#### SOD-like Activity test

SOD-like activity was evaluated the ability of the compounds to mimic the catalytic function of SOD, which converts the superoxide anion (O₂•⁻) into molecular oxygen (O₂) and hydrogen peroxide (H₂O₂) [33]. The method is based on the inhibition of pyrogallol autoxidation by superoxide radicals, as originally described by Marklund and Marklund [34]. Since pyrogallol autoxidation is promoted by superoxide anions, compounds with SOD-like activity reduce the rate of this reaction.

The reaction mixture was incubated for 3 min with vehicle (DMSO) as the control condition, selenocyanates **1–5** at final concentrations of 10 or 200 µM, or the positive antioxidant control (AA, Cf: 100 µM), in Tris–HCl buffer containing pyrogallol. The rate of pyrogallol autoxidation was monitored spectrophotometrically by measuring absorbance at 412 nm for 2 min. Results were expressed as the change in absorbance per minute.

#### Cerebral RS Measurement

This assay investigated whether selenocyanate compounds reduce RS levels in brain tissue using 2′,7′-dichlorofluorescein diacetate (DCFH-DA), as previously described by Lebel et al. [35]. DCFH-DA is a non-fluorescent probe that readily permeates biological membranes and is hydrolyzed intracellularly to dichlorodihydrofluorescein (DCFH). Upon oxidation by RS, DCFH is converted into the highly fluorescent dichlorofluorescein (DCF), allowing sensitive detection of RS generation.

Brain tissue was homogenized in 50 mM Tris–HCl buffer (pH 7.4) at a 1:10 (w/v) ratio and centrifuged at 2500 rpm for 10 min to obtain the supernatant (S_1_), which was used for assay. Aliquots of S_1_ (25 µL) were incubated for 30 min with vehicle (DMSO) as the control condition, selenocyanates (**1–5**) at final concentrations of 10 or 200 µM, or the positive control (BHT, 200 µM). Levels of RS was induced by the addition of ferrous sulfate (FeSO₄, Cf: 0.1 µM) and hydrogen peroxide (H₂O₂, Cf: 2.83 µM) in Tris–HCl buffer. RS production was quantified using DCFH-DA (5 µM), and fluorescence was measured at an excitation wavelength of 488 nm and an emission wavelength of 520 nm. Results were expressed as arbitrary fluorescence units (AU).

#### MAO Activity

Brain tissue was processed to obtain a mitochondria-rich fraction according to the method described by Soto-Otero et al. [36]. The mitochondria-rich samples were incubated with assay buffer at 37 °C for 5 min, in the presence of either pargyline (Cf: 0.25 µM), a selective MAO-B inhibitor, or clorgyline (Cf: 0.25 µM), a selective MAO-A inhibitor. Subsequently, compounds **1–5** at final concentrations of 10 or 200 µM, vehicle (DMSO, as the control condition), or the positive controls pargyline (Cf: 0.25 µM, MAO-A) or clorgyline (Cf: 0.1 µM, MAO-B) were added, followed by incubation for 10 min at 37 °C. The reaction was initiated by the addition of the substrate quinuramine (Cf: 90 µM for the MAO-A assay and 60 µM for the MAO-B assay) and allowed to proceed for 30 min at 37 °C.

The reaction was terminated by the addition of 10% trichloroacetic acid, and the samples were centrifuged for 5 min at 13,000 rpm. The supernatant was collected, followed by the addition of sodium hydroxide (1 M). Fluorescence was measured using a fluorimeter with excitation at 315 nm, emission at 380 nm. Protein content was determined by the Bradford method [37], and MAO activity was expressed as nmol of 4-hydroxyquinoline/mg of protein/minute.

#### AChE Assay

The AChE catalyzes the hydrolysis of acetylthiocholine, generating thiocholine, which subsequently reacts with 5,5′-dithiobis(2-nitrobenzoate) (DTNB) to form the yellow anion 5-thio-2-nitrobenzoate (TNB²⁻). For sample preparation, brain tissue was homogenized in a 1:10 (w/v) ratio with Medium I buffer (0.32 M sucrose, 5 mM Tris-HCl, 0.1 mM EDTA), followed by centrifugation at 3,000 rpm for 10 min. The resulting supernatant (S_1_) was collected, and the protein concentration was determined according to the Bradford method [36].

For the enzymatic assay [38], vehicle (DMSO) as the control condition, positive control (rivastigmine, Cf: 200 µM), or selenocyanate compounds **1–5** (Cf: 5 to 20 µM) were added to each tube, along with 0.1 M Tris-HCl buffer (pH 7.4). Subsequently, an aliquot of S_1_ was added and the samples were pre-incubated at 25 °C for 2 min. The reaction was initiated by the addition of DTNB (Cf: 0.23 mM) and acetylthiocholine iodide (ASChI, Cf: 0.744 mM). Absorbance was monitored at 412 nm using a spectrophotometer, with 0.1 M Tris-HCl buffer (pH 7.4) as a blank. Measurements were recorded every 30 s for a total period of 2 min. Enzyme activity was expressed in µmol/h/mg of protein.

### In Vivo Assay

Following the in vitro analyses, the compound **4** was selected for acute oral toxicity assessment based on its performance in the previously conducted biological assays (antioxidant effects, MAO and AChE inhibition). Additionally, based on its enzyme inhibition profile, particularly against AChE, compound **2** was also selected.

#### Acute Oral Toxicity

Acute oral toxicity was assessed according to OECD guideline 423 [39]. The study was conducted using adult female Swiss mice as suggested by Guideline 423, which were acclimated under standard biothermic conditions prior to the experiment. Females are preferably employed due to their slightly higher sensitivity to toxicological effects observed in historical datasets. The animals were fast for 4 h before oral treatment administration, with free access to water.

Compounds 2 and 4 were administered orally by gavage in separate dose groups (*n* = 6 per group), starting at 50 mg/kg body weight and a fixed volume of 10 mL/kg. Control animals received canola oil as the vehicle. After administration, the animals were closely observed for clinical signs of toxicity, behavioral changes, and mortality during the first 24 h, with special attention during the initial 4 h post-administration. Subsequently, daily observations were carried out for a total period of 14 days, during which body weight, food intake, and water consumption were monitored. In the absence of mortality or severe toxic effects at the initial dose, a subsequent group of animals received a higher dose of 300 mg/kg body weight, following the same experimental procedures and observation schedule.

The use of OECD Guideline 423, up to a dose of 300 mg/kg, was based on an experimentally feasible administration level, taking into account the common physicochemical limitations faced by synthetic organoselenium compounds, particularly their lipophilicity (moderate/high), while ensuring animal safety and enabling reliable toxicological acute screening within a relevant dose range. Thus, this experiment enables identification of tolerated doses, guiding the selection of safe levels for acute pharmacological studies and future investigations.

At the end of the observation period, the animals were subjected to the open field test (OFT) and subsequently anesthetized with isoflurane for blood collection by cardiac puncture to obtain plasma for biochemical analyses. After blood collecting, the animals were euthanized. All procedures were conducted in accordance with ethical guidelines for the care and use of laboratory animals.

#### Open Field Test (OFT)

The OFT was conducted to evaluate the locomotor activity and exploratory behavior of mice as part of a toxicity assessment. The assay was conducted in a square open field box, measuring 40 × 40 × 30 cm and subdivided into equal quadrants on the floor to facilitate behavioral scoring. The apparatus was kept in a soundproof room under controlled lighting and temperature conditions.

According to the Walsh and Cummins protocol [40], the mice were placed individually in the center of the arena and allowed to freely explore the apparatus during a predetermined observation period of 5 min. Locomotor activity was assessed by recording the number of crossings between nine quadrants, while exploratory behavior was assessed by the parameter of rearing on the hind legs. Between each test, the apparatus was carefully cleaned with a 20% ethanol solution to eliminate olfactory cues. The OFT was performed at the end of the treatment period, and the results were used to identify possible neurobehavioral changes associated with exposure to the compounds, thus contributing to the overall toxicological profile.

#### Biochemical Parameters

Ex vivo analyses of alanine aminotransferase (ALT) and aspartate aminotransferase (AST) activities (markers of liver function), as well as urea (kidney function marker) and protein levels, were performed in blood plasma. ALT, AST, and urea were analyzed using commercial kits (LabTest^®^), following the manufacturer’s instructions. ALT and AST results were expressed in international units (IU), whereas urea levels were expressed in mg/dL.

Protein levels were determined using the Bradford method [37]. Absorbance was measured at 595 nm using a spectrophotometer, and protein concentrations were calculated from a standard curve generated with bovine serum albumin (BSA), with results expressed in mg/mL.

Cerebral lipid peroxidation was determined by quantifying thiobarbituric acid-reactive substances (TBARS) according to the method of Ohkawa et al. [41]. Brain tissues were homogenized in 50 mM Tris–HCl buffer (pH 7.4) at a 1:5 (w/v) ratio, and the homogenates were centrifuged at 900 × g for 10 min. The supernatant obtained was collected for subsequent analysis. An aliquot of the supernatant was incubated with a reaction solution containing 0.8% thiobarbituric acid, 8.1% sodium dodecyl sulfate, and acetic acid (pH 3.4) at 95 °C for 2 h. After cooling to room temperature, absorbance was recorded at 532 nm using a spectrophotometer. Lipoperoxidation concentrations were expressed as nmol of malondialdehyde (MDA) per mg of protein (nmol MDA/mg protein).

### Statistical Analysis

The data were analyzed using GraphPad Prism software (version 8.2.0). For the in vitro assays, statistical comparisons were performed using one-way analysis of variance (ANOVA), followed by Tukey’s multiple comparisons post hoc test. The homogeneity of variances was verified using the Brown-Forsythe test. For the in vivo assays, data were analyzed using an unpaired t test, one-way ANOVA followed by Dunnett’s post hoc test or two-way ANOVA followed by Bonferroni post hoc test, as appropriate. Finally, for the ex vivo assays, data were analyzed using an unpaired t test or one-way ANOVA followed by Bonferroni post hoc test, as appropriate. Results are expressed as mean ± standard error of the mean (SEM), and differences were considered statistically significant when *p* ≤ 0.05.

## Results

### In Silico Assays

#### Toxicological and Pharmacokinetic Insights from ADME/T Predictions

The ADME/T profile of selenocyanates (**1–5**) was predicted in Table 1. For absorption, the compounds showed positive Caco-2 permeability, with values ranging from 1.170 to 1.234 log; according to pkCSM criteria, compounds with predicted values above 0.90 are considered to exhibit high intestinal Caco-2 permeability. The predicted human intestinal absorption ranged from 89.904% to 100%; in the pkCSM model, compounds with values below 30% are considered to exhibit low intestinal absorption. In terms of distribution, compounds presented logBB values ranging from − 0.285 to − 0.120, indicating low permeability across the blood-brain barrier (BBB) according to the reference threshold (logBB < 0.3). Consistently, predicted logPS values ranged from − 2.950 to − 2.858, indicating values below the reference threshold (< -2.0) and suggesting limited penetration into the CNS.

**Table 1:** Predicted ADME/T properties of selenocyanates

		Compounds					
	Property	1	2	3	4	5	Unit
Abs.	Caco-2 Permeability	1.170	1.177	1.234	1.226	1.216	log Papp; log cm/s
Abs.	Intestinal Absorption (human)	100.000	100.000	89.904	89.962	90.071	Percentage (%)
Dist.	BBB permeability	-0.285	-0.251	-0.120	-0.132	-0.124	logBB
Dist.	CNS permeability	-2.950	-2.931	-2.862	-2.862	-2.858	logPS
Met.	CYP2D6 substrate	No	No	No	No	No	Yes/No
Met.	CYP3A4 substrate	No	No	No	No	No	Yes/No
Met.	CYP1A2 inhibitor	No	No	Yes	Yes	Yes	Yes/No
Met.	CYP2C19 inhibitor	No	No	No	No	No	Yes/No
Met.	CYP2C9 inhibitor	No	No	No	No	No	Yes/No
Met.	CYP2D6 inhibitor	No	No	No	No	No	Yes/No
Met.	CYP3A4 inhibitor	No	No	No	No	No	Yes/No
Exc.	Total Clearance	1.446	1.532	1.703	1.688	1.685	log(CLtot)
Tox.	AMES toxicity	No	No	No	No	No	Yes/No
Tox.	Oral Rat Acute Toxicity (LD_50_)	2.327	2.334	1.857	1.829	1.717	(mol/kg)
Tox.	Oral Rat Chronic Toxicity (LOAEL)	2.186	2.154	2.371	2.385	2.435	Log(mg/kg_ bw/day)
Tox.	Hepatotoxicity	No	No	No	No	No	Yes/No

Abbreviations - *Abs* Absorption, *Dist* Distribution, *Met* Metabolism, *Exc* Excretion, *Tox* Toxicity, *BBB* Blood-Brain Barrier, *CNS* Central Nervous System, *CYP* Cytochrome P450, *LD*_50_ Lethal Dose 50%, *LOAEL* Lowest Observed Adverse Effect Level

Regarding metabolism, compounds **1** and **2** were predicted not to inhibit major cytochrome P450 isoforms, whereas compounds **3**, **4**, and **5** showed inhibitory activity exclusively against CYP1A2. None of the compounds were predicted to inhibit CYP2D6 and CYP3A4 substrates. For excretion, the predicted total clearance ranged between 1.446 and 1.703 log (CLtot). In the toxicity assessment, four parameters were analyzed: AMES mutagenicity, oral rat acute toxicity (LD_50_), oral rat chronic toxicity (LOAEL), and hepatotoxicity. All selenocyanates were predicted to be non-mutagenic according to the Ames test prediction model. The LD_50_ values ranged from 1.717 to 2.334 mol/kg. The LOAEL values ranged from 2.154 to 2.435 log(mg/kg_bw/day). None of the compounds were predicted to be associated with hepatotoxicity (Table 1).

#### Molecular Docking Predicted Potential Interactions with MAO-A, MAO-B, and AChE

Molecular docking analyzes were conducted for selenocyanates (**1–5**) against three neurochemical targets: MAO-A, MAO-B, and AChE. The predicted binding energies revealed affinities across the proteins (Table 2). Compounds displayed favorable interactions with MAO-A and MAO-B, with binding energies ranging from − 3.996 to − 6.899 kcal/mol, suggesting stable and binding. For AChE, the predicted binding energies ranged from − 4.091 to − 6.767 kcal/mol, also indicating predicted ligand–enzyme interactions. These results suggest that selenocyanates may act as potential inhibitors of key enzymes involved in neurotransmitter metabolism, in agreement with their observed in vitro activities. Molecular docking scores for positive controls clorgyline, pargyline and rivastigmine were − 7.104, -6.217, and − 6.367, respectively (Table 2).

**Table 2:** Molecular docking scores of selenocyanate compounds with enzymes MAO-A, MAO-B and AChE

Compound	Receptor enzyme		
	MAO-A (kcal/mol)	MAO-B (kcal/mol)	AChE (kcal/mol)
1 2 3 4 5	-4.327 -4.136 -6.270 -6.899 -6.568	-4.162 -3.996 -6.057 -6.514 -6.513	-4.091 -4.094 -6.463 -6.767 -6.748
Clorgyline	-7.104	-	-
Pargyline	-	-6.217	-
Rivastigmine	-	-	-6.367

Abbreviations – *MAO* monoamine oxidase, *AChE* acetylcholinesterase

The analysis of the most favorable binding conformations of the compounds within the target receptors enables the identification of key amino acid residues involved in ligand–enzyme interactions, as well as the characterization of the interaction types and their corresponding binding energies.

**Fig. 3:**
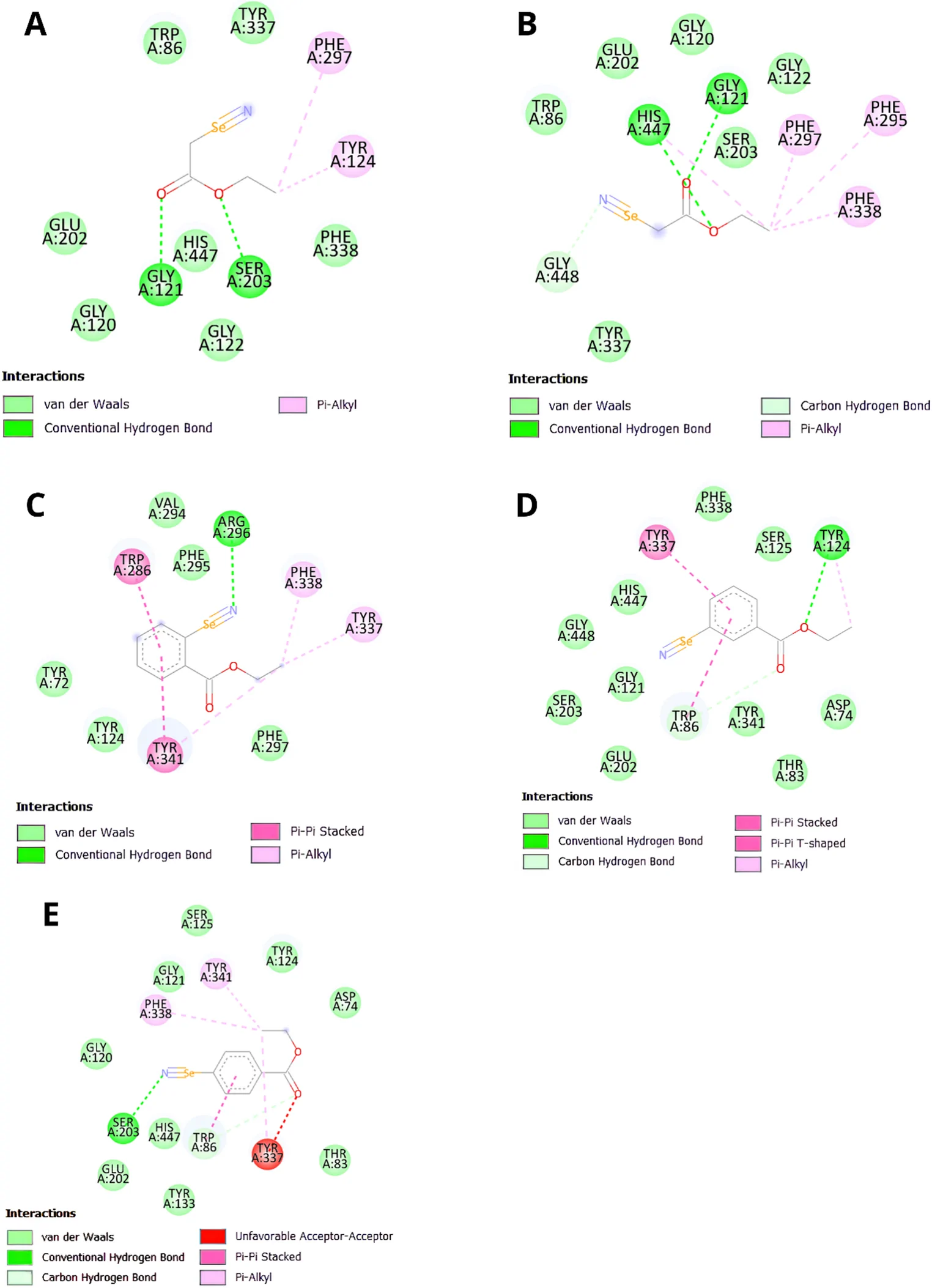
Interaction map showing the 2D binding interactions of compounds 1 (**A**), 2 (**B**), 3 (**C**), 4 (**D**), and 5 (**E**) with the enzyme AChE

Molecular docking analysis of AChE revealed that, with respect to Van der Waals interactions, the most prominent active site residues were GLU202 (compounds **1**, **2**, and **4**), GLY120 (compounds **1**, **2**, and **5**), HIS447 (compounds **1**, **4**, and **5**), GLY121 (compounds **4** and **5**), and SER203 (compounds **2** and **4**), TRP86 (compounds **1** and **2**). Notably, GLY121 and SER203 also established conventional hydrogen bonds with compounds** 1** and **2**, and with compounds **1** and **5**, respectively, as illustrated in Fig. 3 and S11–S15.

Carbon–hydrogen bonds were observed between compounds **4** and **5** and the TRP86 active site. Regarding alkyl interactions, compounds **2**, **3**, and **5** interacted with PHE338, which also exhibited Van der Waals interactions with compound **1**. In addition, amide–π stacking interactions were identified for compound** 3** with TRP286 and TYR341, and for compound **4** with TYR337 (Fig. 3 and S11–S15).

The positive control rivastigmine revealed important interactions with the amino acids SER203, TRP86, PHE338, TRP286 and HIS447 within of active site (Figure S16).

**Fig. 4:**
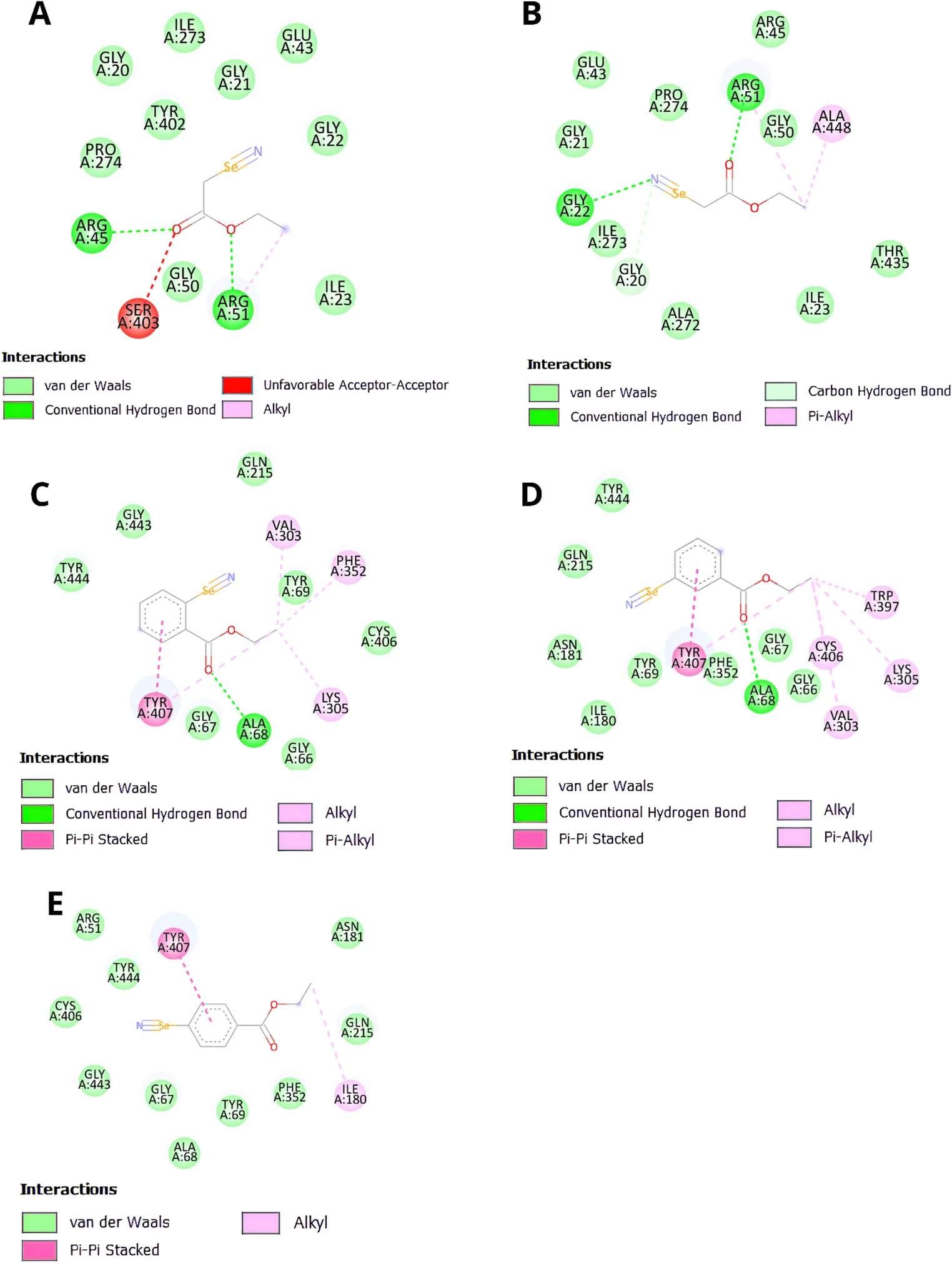
Interaction map showing the 2D binding interactions of compounds 1 (**A**), 2 (**B**), 3 (**C**), 4 (**D**), and 5 (**E**) with the enzyme MAO-A

Molecular docking of MAO-A identified GLN215, TYR69, TYR444, and GLY67 as the main residues involved in Van der Waals interactions with compounds **3**–**5**. Conventional hydrogen bonds were formed between ARG51 and compounds **1** and **2**, and between ALA68 and compounds **3** and **4** (Fig. 4 and S17–S21). Carbon–hydrogen bonding was observed only for compound **2** at GLY20. Alkyl interactions involved compound **2** with ALA448, compound **3** with LYS305, VAL303, and PHE352, compound **4** with CYS406, TRP397, LYS305, and VAL303, and compound **5** with ILE180. In addition, compound **3**–**5** exhibited amide–π stacking interactions with TYR407 (Fig. 4 and S17–S21).

 The positive control also formed interactions with important residues active site such as TYR407, TYR69, TYR444 and PHE352 (Figure S22).

**Fig. 5:**
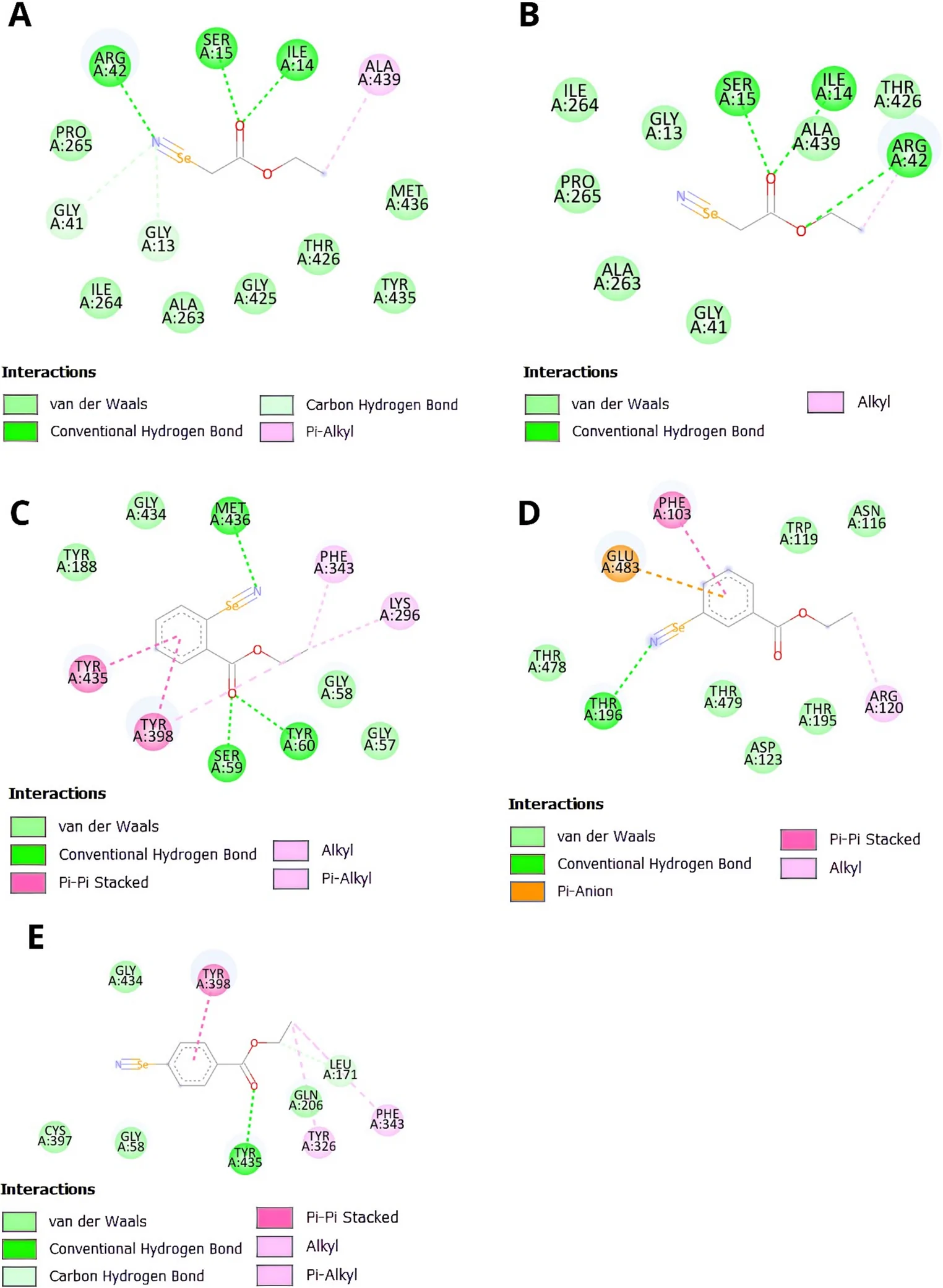
Interaction map showing the 2D binding interactions of compounds 1 (**A**), 2 (**B**), 3 (**C**), 4 (**D**), and 5 (**E**) with the enzyme MAO-B.

Molecular docking analysis of MAO-B revealed that compounds **1** and **2** established Van der Waals interactions with the active site residues ILE264, PRO265, ALA263, and THR426. Similarly, compounds **3** and **5** interacted with GLY434 and GLY58as illustrated in Fig. 5.

 Regarding conventional hydrogen bonds, compounds **1** and **2** interacted with ILE14, SER15, and ARG42, whereas compound **3** formed hydrogen bonds with TYR60, SER59, and MET436. Compounds **4** and **5** exhibited single hydrogen-bond interactions with THR196 and TYR435, respectively. Carbon–hydrogen bonds were detected only for compounds **1**, involving GLY13 and GLY41, and for compound **5** with LEU171 (Fig. 5 and S23–S27).

 Alkyl interactions were identified for compounds **3** and **5** with PHE343; additionally, compound **5** interacted with TYR326 and compound** 3** with LYS296. Compounds **1** and **4** showed alkyl interactions with ALA439 and ARG120, respectively. Further stabilizing interactions included π–π interactions with TYR398 (compounds **3** and **5**), TYR435 (compound **3**), and PHE103 (compound **4**), as well as a π–anion interaction between GLU483 and compound **4** (Fig. 5 and S23–S27).

 The positive control showed interactions with amino acids in the active site, such as TYR435 and TYR60 (Figure S28).

###  In Vitro Assays

####  Antioxidant Effects of Selenocyanate Compounds

**Fig. 6:**
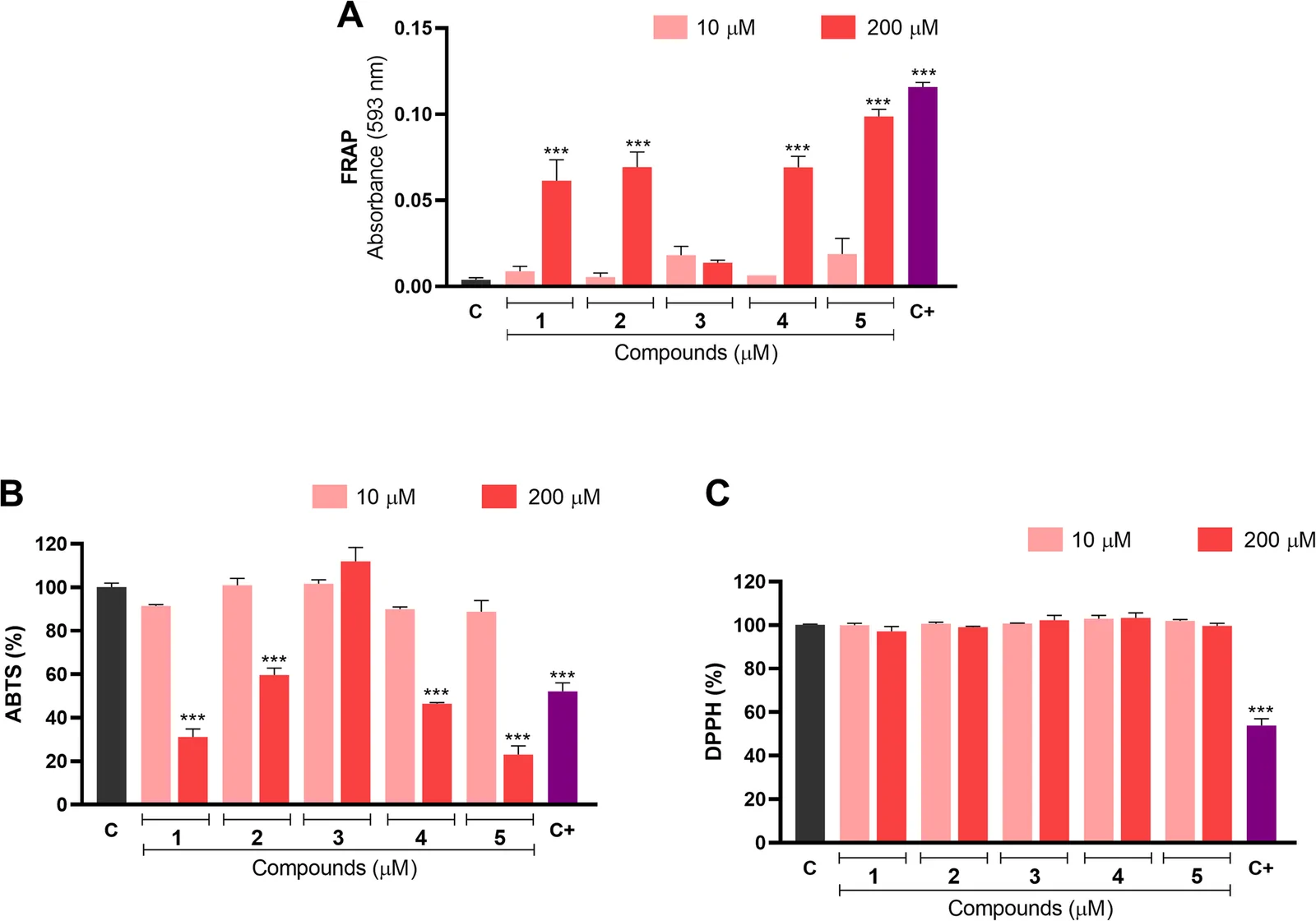
Effects of the selenocyanate compounds **1** to **5** (10 and 200 µM) on FRAP (**A**), ABTS (**B**), and DPPH (**C**) assays. One-way ANOVA was used with Tukey’s post hoc multiple comparisons test. ***p < 0.001 vs. control (**C**). All controls received the vehicle of selenocyanates (DMSO). C+ refers to the positive controls: ascorbic acid (AA, 10 µM). Data are expressed as mean ± standard error of the mean (SEM).

The results of FRAP, ABTS, and DPPH are demonstrated in Fig. 6.

 The effects of selenocyanate compounds **1–5** on FRAP are presented in Fig. 6A. One-way ANOVA indicated a significant difference among the experimental groups [F(11,24) = 46.25; p < 0.0001]. Tukey’s post hoc analysis demonstrated that compounds **1**, **2**, **4**, and **5** at the concentration of 200 µM showed significant differences compared with the control group. As expected, the positive control AA (10 µM) exhibited antioxidant activity, validating the experimental procedure.

Figure 6B shows the results of the ABTS radical scavenging assay. ANOVA revealed a significant difference among the experimental groups [F(11,24) = 78.33; p < 0.0001]. Tukey’s post hoc test indicated that compounds **1**, **2**,** 4**, and **5** at the concentration of 200 µM differed significantly from the control group (p < 0.001). The effectiveness of the positive control AA (10 µM) confirmed the validity of the assay.

The result of the DPPH radical is shown in Fig. 6C. One-way ANOVA showed a statistically significant difference among the experimental groups [F(11,24) = 75.03; p < 0.0001]. However, Tukey’s post hoc analysis revealed that none of the selenocyanate derivatives differed significantly from the control group. In contrast, the positive control AA (10 µM) showed significant activity, confirming the suitability of the method.

#### Some Selenocyanate Compounds Enhance Antioxidant Defense

**Fig. 7:**
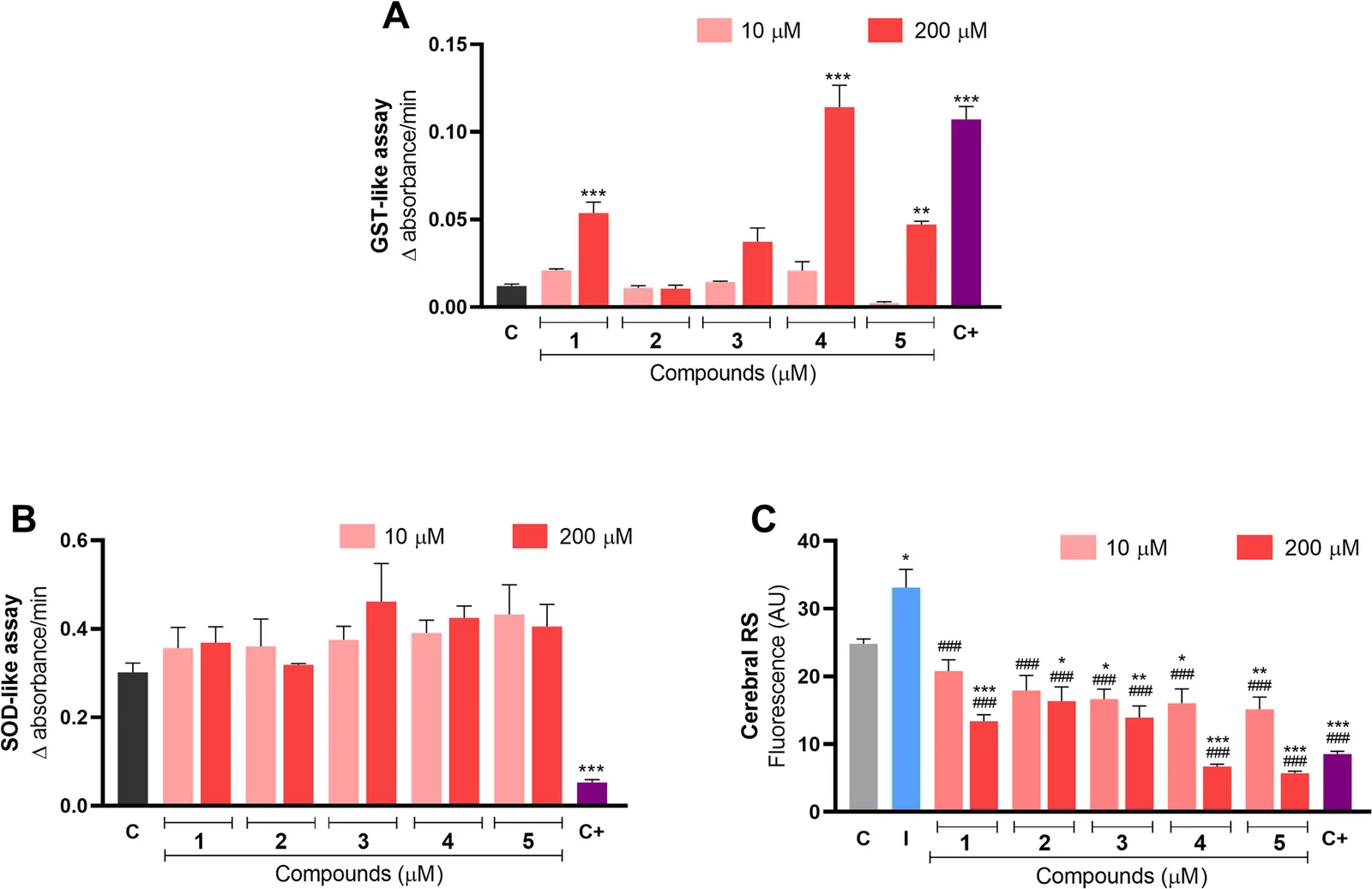
Effects of the selenocyanate compounds **1** to **5** (10 and 200 µM) on GST-like activity (**A**), SOD-like activity (**B**) assays and RS levels (**C**). One-way ANOVA followed by Tukey’s post hoc test. *p < 0.05; **p < 0.01 and ***p < 0.001 vs. control (**C**). Controls in the GST-like and SOD-like assays as well as induced group (I) in the RS assay received the vehicle of selenocyanates (DMSO). #p < 0.05; ##p < 0.01 and ###p < 0.001 vs. induced group. C+ refers to the positive controls: (**A**) (PhSe)2 (25 µM), (**B**) ascorbic acid (AA, 100 µM) and (**C**) butylated hydroxytoluene (BHT, 100 µM). (I) refers to ferrous sulfate/hydrogen peroxide-induced group in RS level (C). Data are expressed as mean ± standard error of the mean (SEM).

Figure 7 illustrates the behavior of the selenocyanate derivatives in the GST-like, SOD-like activity assays and RS levels.

The effects of the compounds **1–5** on GST-like assay are shown in Figure 7A. One-way ANOVA revealed a significant difference among the experimental groups [F(11, 24) = 48.11; p < 0.0001]. However, post hoc analysis using Tukey’s test indicated that the compounds (**1**, **4** and **5**) exhibit a significant difference at concentration 200 µM compared with control group, i.e., they can conjugate GSH to the electrophilic substrate CDNB. The positive control (PhSe)2 (25 µM) was effective as GST-like agent, validating the findings.

The actions of the compounds on SOD-like assay are demonstrated in Fig. 7B. One-way ANOVA revealed a significant difference among the experimental groups [F(11,24) = 5.391; p < 0.0003]. However, post hoc analysis using Tukey’s test indicated that the compounds did not show significant effects in comparison with the control group, that is none of the compounds were able to reduce the rate of pyrogallol autoxidation and, therefore, were unable to neutralize the superoxide anion. As expected, the positive antioxidant control AA (100 µM) significantly reduced the absorbance compared to the control, validating the findings.

Figure 7C illustrates the effects of compounds **1–5** on cerebral RS levels. One-way ANOVA revealed a statistically significant difference among the experimental groups [F(12,52) = 20.78; p < 0.0001]. Tukey’s post hoc test showed that ferrous sulfate and hydrogen peroxide combination, used as an RS inducer, significantly increased brain RS levels compared with the control group. Compared to the induced group, all compounds significantly reduced RS levels at both 200 µM and 10 µM. Furthermore, all compounds at a concentration of 200 µM, differed significantly from the control group, as did compounds **4** and **5** at a concentration of 10 µM. The positive antioxidant control BHT (100 µM) was effective, supporting the reliability of the assay. 

####  Selenocyanate Compounds Inhibit Cerebral MAO Isoforms and AChE Activity

**Fig. 8:**
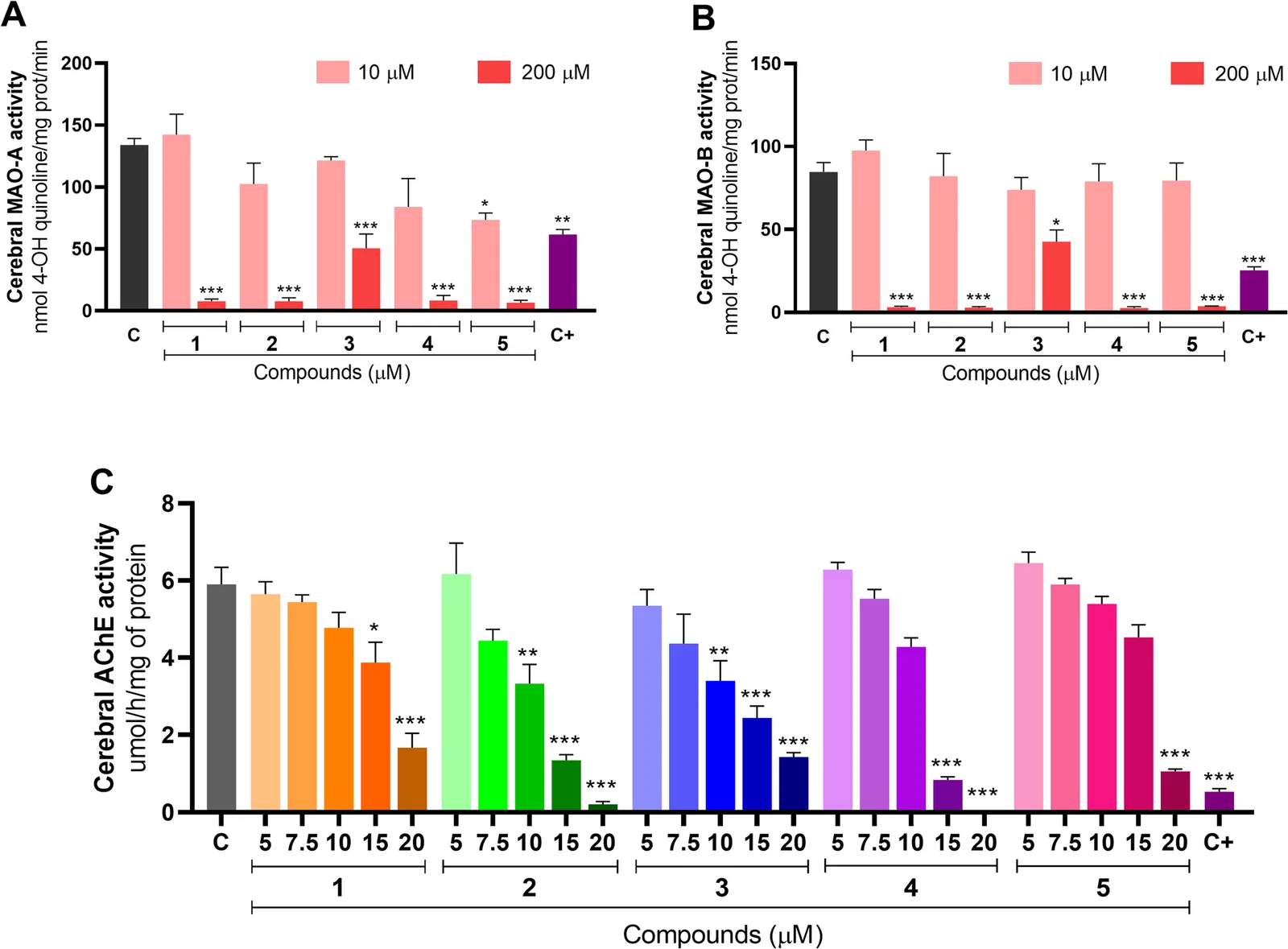
Effects of selenocyanate compounds **1** to **5** on monoamine oxidase A (**A**) and B (**B**) (10 and 200 µM) or acetylcholinesterase activity (**C**) (5–20 µM). One-way ANOVA followed by Tukey’s post hoc test. *p < 0.05; **p < 0.01; ***p < 0.001 vs. control (**C**). All controls received the vehicle of selenocyanates (DMSO). C+ refers to the positive controls, pargyline 0.25 µM (**A**), clorgyline 0.1 µM (**B**) and rivastigmine 200 µM (**C**). Data are expressed as mean ± standard error of the mean (SEM).

Figure 8 illustrates the effects of the selenocyanate derivatives on the enzymatic activity of MAO isoforms (MAO-A and MAO-B) and AChE in mouse brain tissue in vitro.

The effects of the selenocyanate compounds on activity of MAO isoforms are shown in Figure 8A (MAO-A) and 8B (MAO-B). One-way ANOVA revealed a significant difference among experimental groups for both MAO-A [F(11,24) = 24.18; p < 0.0001] and MAO-B [F(11,24) = 29.47; p < 0.0001]. In Tukey’s post hoc test, MAO-A showed a significant difference for compound 5 in 10 µM and for all compounds (1-5) at concentration 200 µM, while MAO-B showed a significant difference for compounds **3** and **4** at 10 µM and for all compounds (**1-5**) at concentration 200 µM, compared to the control group. The positive controls pargyline (B) and clorgyline (C) validate the results.

The results concerning the effects of the compounds on AChE activity are presented in Fig. 8C. The one-way ANOVA indicated a statistically significant difference among the experimental groups [F(26,54) = 34.96; p < 0.0001]. Subsequent Tukey’s post hoc analysis revealed that all compounds tested exhibit significant differences with the control group. Compounds **1** and **4** exhibited AChE inhibitory activity from a concentration of 15 µM, with IC50 values of 16.45 and 11.55 µM, respectively. Compounds **2** and **3** were effective at concentrations as low as 10 µM, displaying IC50 values of 10.57 and 12.11 µM, respectively. Compound **5** showed significant activity from 20 µM, with an IC50 value of 17.03 µM. Overall, the estimated inhibitory potency against AChE decreased in the following order: Compound 2 > Compound 4 > Compound 3 > Compound 1 > Compound 5, according to their IC50 values. Experimentally, at the highest concentration tested (20 µM), compounds 2 (96.61% of inhibition) and 4 exhibited the greatest inhibitory potency (100%). The positive control of rivastigmine (200 µM, a well-known AChE inhibitor) demonstrated effectiveness, confirming the validity of the experimental findings. Based on the findings, we selected compounds 2 and 4 for in vivo safety profile evaluation.

###  Toxicity

#### Supplementary Predicted Physicochemical Properties of the Selected Selenocyanates

For the selected compounds (compound **2** ‒ aliphatic; compound **4** ‒ aromatic), supplementary ADMET predictions, including metabolic stability, lipophilicity, and pKa, are presented in Table S1. Both compounds were predicted to be highly susceptible to biotransformation by human liver microsomes (HLM) and were classified as metabolically unstable (≤ 30 min), with compound **4** being slightly more metabolically unstable than compound **2** (0.999 and 0.983, respectively). Regarding physicochemical properties, compound **2** (aliphatic) exhibited lower lipophilicity (LogP = 0.836; LogD = 0.647) and higher pKa values (acidic pKa = 8.385; basic pKa = 4.825) compared to compound **4** (aromatic) (LogP = 2.422; LogD = 2.356; acidic pKa = 2.822; basic pKa = 8.368 (Table S1).

#### Acute Oral Toxicity Protocol

Safety assessments typically employ relatively high doses to identify potential toxic effects in animals. Accordingly, an acute oral toxicity assay was conducted to evaluate compounds **2** and **4**, selected based on their in vitro activity, starting with an initial dose of 50 mg/kg, followed by testing at 300 mg/kg. No mortality and no observable alterations in physical signs were detected at 50 mg/kg for both compounds. Specifically, the animals treated with 50 mg/kg showed no evidence of piloerection, tremors, convulsions, salivation, abnormal posture, respiratory distress, lethargy, or other behavioral and neurological abnormalities. However, at 300 mg/kg, compound **2** induced mortality (*n* = 3; set 1), leading to discontinuation of the protocol for this group.

**Fig. 9:**
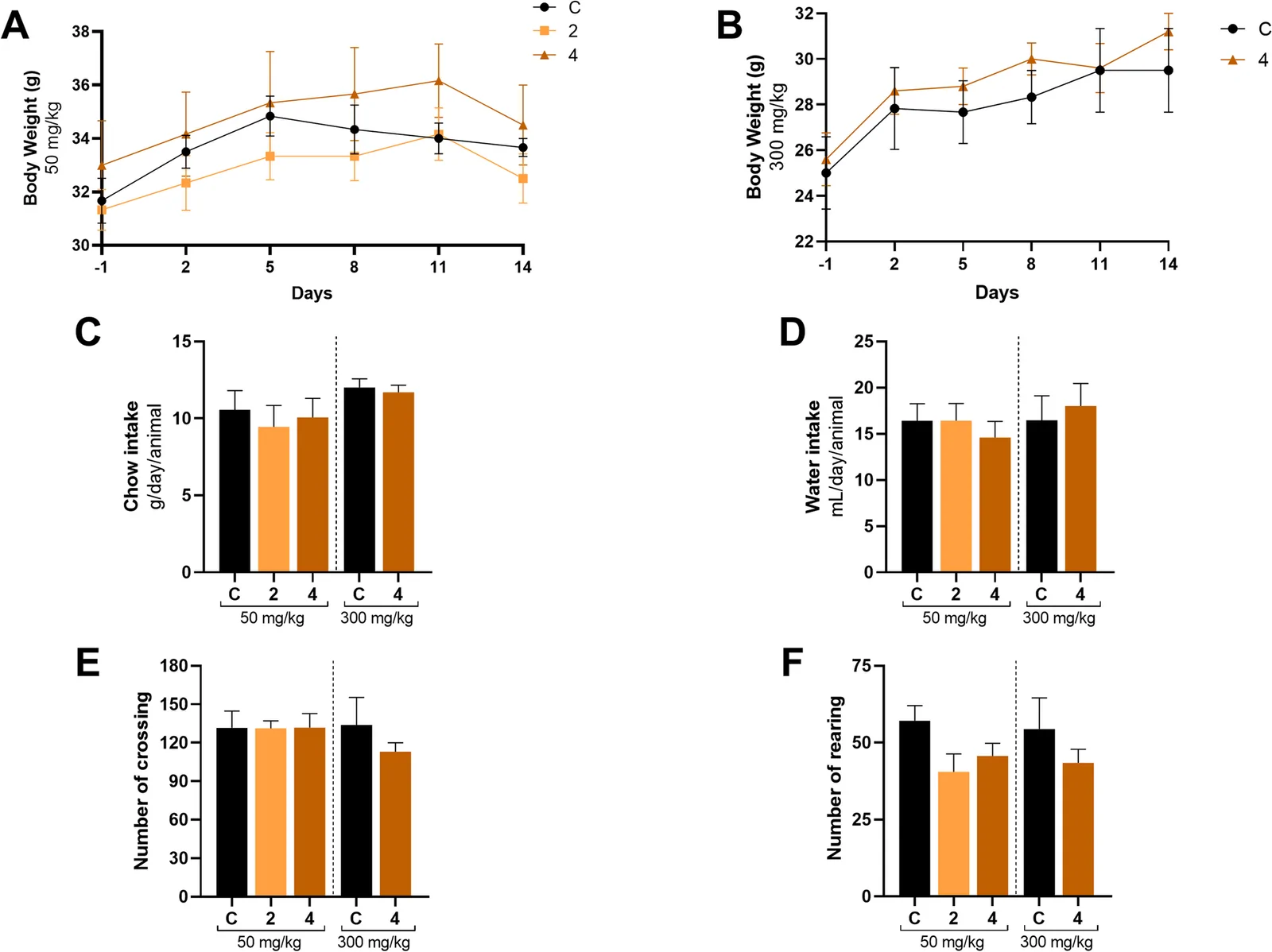
Evaluation of physical and locomotor parameters following administration of compounds **2** and **4** (50 or 300 mg/kg). The control group (**C**) received canola oil, which was used as the vehicle for the in vivo administration of selenocyanates. Body weight at 50 mg/kg (**A**), body weight at 300 mg/kg (**B**), food intake (**C**), water intake (**D**), number of crossings (**E**), and number of rearing in the open field test (OFT) (**F**). For panels A, B, E, and F individual animal data (n = 6 per group) were analyzed. For panels C and D, average food and water intake per animal were recorded every three days throughout the experimental period. Data are expressed as mean ± standard error of the mean (SEM).

During toxicity studies, monitoring body weight as well as food and water intake is essential for evaluating the effects of test substances on animal health and welfare. Body weight assessed over 14 days did not differ significantly among the experimental groups at either 50 mg/kg [F_(10,90)_ = 0.1003; *p* = 0.9998; Bonferroni’s test] (Fig. 9A) or 300 mg/kg [F_(5,54)_ = 0.1001; *p* = 0.9917; Bonferroni’s test] (Fig. 9B).

Food intake in animals treated with compounds **2** and **4** did not differ from the control group at 50 mg/kg [F_(2,18)_ = 0.1788 *p* = 0.8377; Dunnett’s test], nor for compound **4** at 300 mg/kg [t_(12)_ = 0.4214; unpaired t test], as shown in Fig. 9C. Additionally, water consumption did not differ significantly between treated and control groups at either dose, with no effects observed at 50 mg/kg [F_(2,18)_ = 0.3265; *p* = 0.7256; Dunnett’s test] or at 300 mg/kg [t_(12)_ = 0.4230; unpaired t test], as shown in Fig. 9D.

Finally, the OFT was performed to evaluate locomotor activity and to rule out potential psychomotor alterations associated with acute toxicity. No significant differences were observed in locomotor (Fig. 9E) or exploratory behavior (Fig. 9F) at any of the tested doses. At 50 mg/kg, neither crossings [F_(2,15)_ = 0.0011; *p* = 0.9989] nor rearings [F_(2,15)_ = 2.917; *p* = 0.0851] showed significant changes compared with the control group, according to Dunnett’s post hoc test. Similarly, at 300 mg/kg, no significant differences were detected in crossings [t_(10)_ = 0.9206; unpaired t test] or rearings [t_(10)_ = 0.9946; unpaired t test], as determined by the unpaired t-test. Overall, these findings indicate that the treatments with compound **2** at 50 mg/kg and with compound **4** at 50 and 300 mg/kg did not induce neurobehavioral impairment under the experimental conditions evaluated.

**Fig. 10:**
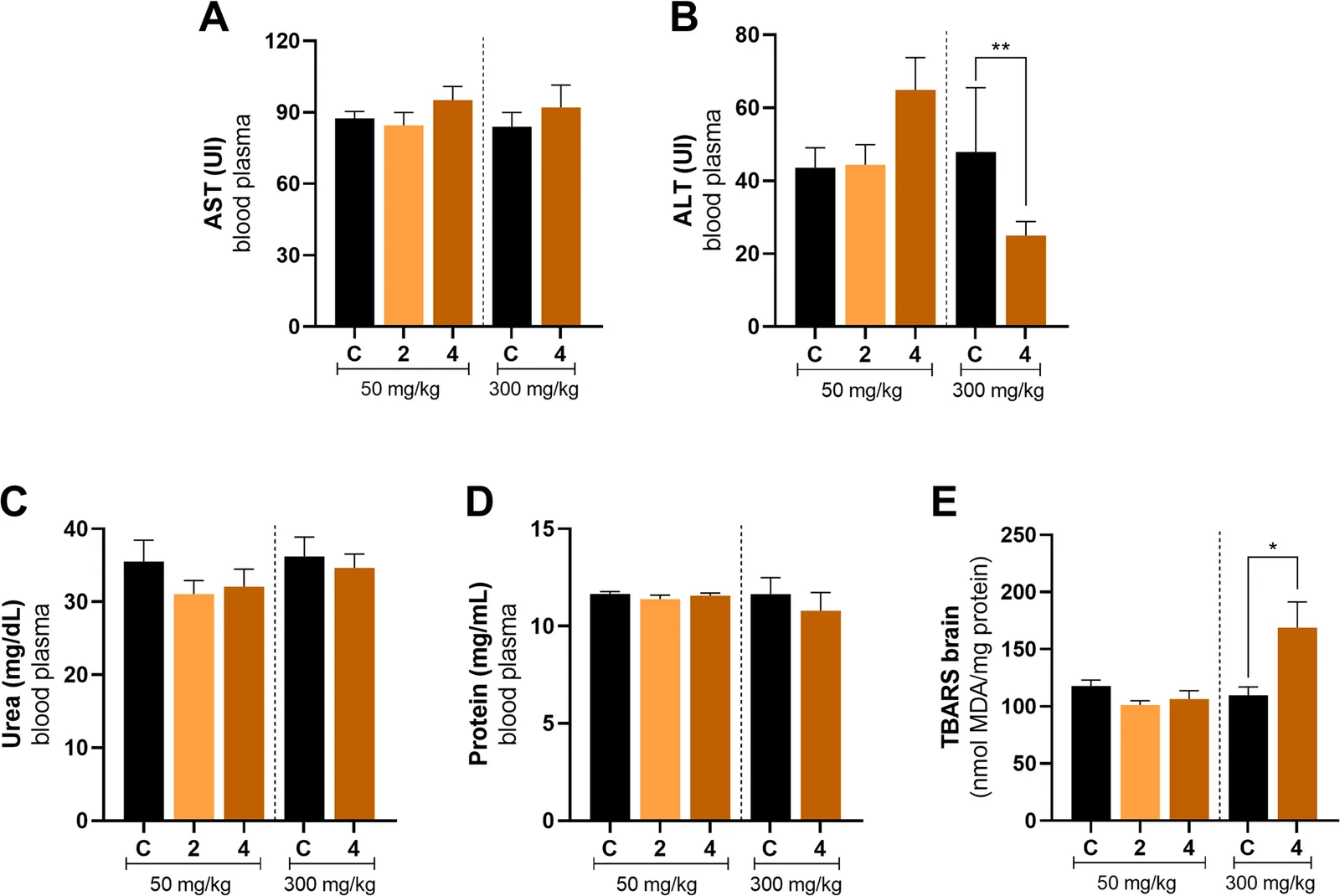
Evaluation of the biochemical parameters AST (A), ALT (**B**), urea levels (**C**) and protein levels (**D**) in blood plasma and cerebral TBARS levels (**E**) of mice treated with compounds 2 and 4 at doses of 50 or 300 mg/kg. For all panels, individual animal data (n = 6 per group) were analyzed. Data are expressed as mean ± standard error of the mean (SEM). Statistical analyses were performed using unpaired t-test; *p < 0.05 and **p < 0.01 vs. control (**C**).

The results of the ex vivo analyses are shown in Fig. 10. AST activity showed no significant difference from the control at any of the doses tested: 50 mg/kg [F(2,15) = 1.288; p = 0.3045; Bonferroni’s test] or 300 mg/kg [t(10) = 0.7289; unpaired t test] (Fig. 10A). Similarly, ALT activity (Fig. 10B) was not significantly altered from the control at the 50 mg/kg dose [F(2,15) = 3.136; p = 0.0728; Bonferroni’s test]. However, at the highest dose (300 mg/kg), a statistically significant effect was observed [t(10) = 1.273; unpaired t test], indicating that ALT activity was significantly reduced compared to the control group at this dose.

Urea levels were also not significantly affected from the control at either dose: 50 mg/kg [F(2,15) = 0.9244; p = 0.4182; Bonferroni’s test] or 300 mg/kg [t(10) = 0.4773; unpaired t test] (Fig. 10C). Likewise, protein levels (Fig. 10D) showed no significant differences relative to the control group at 50 mg/kg [F(2,15) = 0.7424; p = 0.4927; Bonferroni’s test] or 300 mg/kg [t(10) = 0.6708; unpaired t test]. In addition, cerebral TBARS levels showed no significant difference compared to the control group at the 50 mg/kg dose [F(2,15) = 2.302; p = 0.1343; Bonferroni’s test]; however, at 300 mg/kg [t(10) = 2.483; unpaired t test], a statistically significant difference was observed, indicating increased lipid peroxidation at the highest dose tested (Fig. 10E).

###  Discussion

An integrated in silico, in vitro, and in vivo strategy is fundamental for the robust characterization of pharmacological activity, as it enables the connection of computational predictions with experimental and physiological relevance. The present study highlights the bioactive potential of five organic selenocyanate derivatives, considering their biochemical properties and possible pharmacological and toxicological limitations. In this context, the results obtained demonstrated the antioxidant capacity of the selenocyanates through a series of assays exploring different mechanisms of action. in addition to proving safe at a dose of 50 mg/kg in the acute oral toxicity test.

The combined application of complementary biochemical and enzymatic assays enabled a comprehensive and mechanistically informative evaluation of the antioxidant and neuroprotective potential of the investigated compounds. The FRAP assay provided information regarding the reducing power of the molecules, particularly their ability to donate electrons and reduce Fe³⁺ to Fe²⁺ [29]. Compounds **1**, **2**,** 4**, and **5**, at a concentration of 200 µM, exhibited significant ferric reducing capacity, indicating a high electron-donating potential. This property is particularly relevant under conditions of OS, in which redox-active metals may catalyze the formation of highly reactive radicals through Fenton-type reactions [42]. By acting as electron donors, these compounds may stabilize oxidized intermediates and limit radical propagation, thereby attenuating oxidative damage [29].

Complementarily, the ABTS radical scavenging assay confirmed that compounds **1**, **2**, **4**, and **5** (200 µM) are capable of neutralizing pre-formed radicals through mechanisms involving electron transfer (ET) and hydrogen atom transfer (HAT) [43]. The convergence of results obtained from different methodological approaches reinforces the consistency of the observed antioxidant profile. In contrast, no significant activity was detected against the DPPH radical for the evaluated selenocyanates. This finding may be related to the fact that DPPH is more sensitive to direct hydrogen transfer in organic media, whereas the ABTS assay, performed in aqueous medium, exhibits greater versatility and the ability to detect compounds that act predominantly via electron transfer, even when they are less efficient hydrogen donors [44].

The absence of SOD-like activity suggests that the compounds do not directly promote superoxide anion dismutation. Conversely, the GST-like activity observed for compounds **1**, **4**, and **5** (200 µM) highlights their possible role in strengthening detoxification pathways and conjugation reactions, contributing to the maintenance of cellular redox homeostasis [45–47].

Finally, the measurement of RS levels provided direct evidence of the ability of compound **1** (200 µM) and compounds **3–5** (10 and 200 µM) to attenuate oxidative imbalance in biological systems. The observed reduction in RS levels indicates that these compounds may act both through direct neutralization of RS and through interruption of oxidative chain reactions, contributing to the preservation of cellular redox homeostasis [48]. While the FRAP, ABTS, and GST-like assays provide chemical evidence of redox reactivity, RS quantification demonstrates functional activity in a biological context, strengthening the translational relevance of the findings.

The present findings corroborate growing evidence that organoselenium compounds exhibit multifactorial antioxidant activity, dependent on both their chemical structure and the biological system evaluated, which may be partially associated with the redox reactivity of the selenium atom. For instance, Wang et al. [49] demonstrated that chitosan derivatives grafted with selenocyanate functional groups displayed superior antioxidant activity (DPPH scavenging) compared to native chitosan, highlighting the importance of the selenocyanate moiety present in our compounds. These results are consistent with those reported by Frizon et al. [50], who described protective effects of selenocyanates in cultured mouse neurons under oxidative stress, associated with reduced RS production and preservation of the cellular antioxidant system. Additional literature evidence [2] indicates that treatment of mice with diphenylmethyl selenocyanate attenuated hepatic oxidative stress. Moreover, another synthetic organoselenium compound, 1,4-phenylenebis(methylene)selenocyanate, has been shown to induce GSH levels through activation of the Nrf2 pathway [51]. A previous study from our group with selenotetrazoles (lacking the selenocyanate group) [52] showed in vitro antioxidant activity in RS, FRAP, and ABTS assays, albeit with lower potency, and no GST-like activity, unlike the selenocyanates evaluated here. Our results thus support the cytoprotective role of selenocyanate derivatives in maintaining redox homeostasis, highlighting their potential as therapeutic candidates for oxidative stress-related disorders.

MAO is directly related to RS formation, as during the oxidative deamination of monoamines, H₂O₂ is generated as a byproduct, contributing to increased cellular OS. In this context, modulation of MAO activity indicates a potential role in regulating monoaminergic signaling and reducing oxidative byproducts generated during neurotransmitter metabolism [14]. All compounds demonstrated activity at 200 µM in both MAO isoforms.

In agreement with the in vitro analyses, the predictive profile observed for MAO-A demonstrated that compounds **3**, **4**, and **5**, which exhibited more favorable binding energies, established interactions with amino acids located in the active site (TYR444, PHE352, TYR69, TYR407, ASN181, and ILE180) [53], indicating a binding mode consistent with potential enzymatic inhibition. The positive control clorgyline also interacted with residues TYR444, PHE352, TYR69 and TYR407, further reinforcing its potential as an inhibitor. In contrast, compounds **1** and **2**, which presented higher binding energies, did not interact with key catalytic residues, suggesting that their predicted conformations may correspond to peripheral or non-catalytic regions of the enzyme.

A similar trend was observed for MAO-B: compound **2**, with the highest binding energy, did not interact with any amino acid in the active site, whereas compound **1**, with slightly lower energy, formed a single interaction within the active site (TYR435) [22]. Compounds **3**, **4**, and **5**, presenting lower binding energies, established interactions with multiple active-site amino acids (TYR435, PHE343, TYR398, TYR60, PHE103, TRP119, LEU171, TYR326, and TYR343), with TYR435, TYR398 and TYR60 being residues that were also involved in interactions with the positive control pargyline [22, 54]. This suggests that different ligand orientations may still converge toward catalytically relevant regions, also indicating partial agreement between docking scores and functional relevance for this enzyme.

AChE inhibition suggests the capacity to enhance cholinergic neurotransmission [15], which is particularly relevant considering the significant effects observed at low concentrations (< 20 µM). In the molecular docking analysis for AChE, compounds **1** and **2**, which exhibited slightly higher binding energies, interacted with key residues of the active site (SER203 and HIS447) and formed contacts with residues belonging to the peripheral anionic site (PAS) (TRP86, TYR337, TYR124, GLY448, and GLU202) [55].

Interestingly, compound **3**, with lower binding energy, interacted exclusively with residues of the peripheral anionic site (TYR337, TRP286, and TYR124), whereas compounds **4** and **5**, despite exhibiting lower docking energies, established interactions with both catalytic site residues (SER203 and HIS447) and peripheral anionic site residues (TRP86, TYR337, GLY448, ASP74, TYR133, TYR124, and GLU202) [55]. This interaction pattern suggests that binding modes involving both the catalytic gorge and the peripheral anionic site may be more relevant for potential enzymatic inhibition than docking scores alone would indicate. Interactions of TRP86, TYR337, ASP74, TYR124 and TRP286 were also shared by the positive control rivastigmine.

Overall, molecular docking analysis revealed differences in binding energies and interaction profiles with active-site residues among the evaluated compounds. Compounds **4** and **5** consistently exhibited lower binding energies combined with a greater number of interactions with key enzymatic residues, suggesting a higher probability of multitarget activity. In this context, the biological evaluation of AChE and MAO activity (both isoforms) provided relevant information regarding the neuromodulatory potential of the compounds. Together, these findings corroborate a multitarget mechanism of action, integrating antioxidant properties with the modulation of key enzymes involved in neurotransmission.

Our results are consistent with reports in the organoselenium literature. Beyond their antioxidant properties, several compounds have shown preclinical evidence of efficacy as cerebral MAO inhibitors with mouse or rat brains. Bortolatto et al. [56] reported that 2,2′-dithienyl diselenide inhibited both cerebral MAO-A and MAO-B. Similarly, Sampaio [57] demonstrated that 4-organoseleno-isoquinoline derivatives selectively and reversibly inhibited cerebral MAO-B at micromolar concentrations. Interestingly, Elshaflu et al. [58] reported MAO inhibition with preferential selectivity for MAO-B at nanomolar range but using human recombinant membrane-bound MAO. Moreover, both inorganic and organic selenium supplementation in rats reduced brain MAO-B activity ex vivo in adult rats, consistent with their antioxidant effects found [59]. Regarding AChE inhibition, studies by Kumawat et al. [60] and Refaay et al. [61] reported micromolar activity of organoselenium derivatives, supporting the biological relevance of the present compounds; in the latter, a tetrazole-based selenocyanate was the most potent compound. Taken together, these findings confirm the enzyme inhibition potential of organoselenium scaffolds and reinforce their relevance as drug candidates.

In silico analyses, including molecular docking, are limited by their dependence on static protein structures, simplified scoring functions, and incomplete treatment of solvent effects and molecular dynamics, and consequently do not always directly correlate with biological results; therefore, experimental validation remains essential [62].

Based on the in vitro results, the study proceeded with the selection of two compounds (**2** and **4**) for the acute oral toxicity protocol. Compound **2** was selected due to its significant AChE inhibition at concentrations ≥ 10 µM, a determining factor for its selection, in addition to significant MAO reduction. Activity on this enzyme is particularly relevant, since AChE regulates synaptic levels of acetylcholine, a neurotransmitter essential for cognitive processes such as learning and memory. In contrast, compound **4** demonstrated superior performance in the evaluated assays, both in antioxidant assays and regulatory enzymes, exhibiting the most pronounced effects among all tested compounds, reinforcing its promising pharmacological profile and prioritizing it for future investigations.

The predicted ADME/T profile analysis indicated the absence of hepatotoxicity and AMES mutagenicity, suggesting that the compounds are neither genotoxic nor associated with hepatic injury [63]. Notably, the acyclic (linear) aliphatic compounds (**1** and **2**) showed higher predicted LD_50_ values in rats compared to the aromatic derivatives (**3–5**) suggesting reduced acute oral toxicity. In accordance with this structural trend, predicted human intestinal absorption was higher for the linear compounds (**1** and **2**), likely reflecting their simpler structures and lower molecular mass, which favor intestinal permeability [64, 65]. Regarding metabolism, only the aromatic compounds (**3–5**) were predicted to inhibit CYP1A2, an enzyme involved in the hepatic biotransformation of xenobiotics, suggesting a potential risk of metabolic interference and toxicity [66].

Permeability predictions indicated a limited ability of the selenocyanate derivatives to cross the BBB, which may restrict their application in therapeutic strategies requiring direct action in the CNS [67]. Despite the promising neuroprotective effects observed in the present study, the limited permeability of the BBB could emerge as a potential challenge for therapeutic translation. Nanotechnology-based approaches, including lipid nanoparticles, polymeric nanoparticles, and nanoemulsions, have shown considerable potential for enhancing BBB transport and increasing drug accumulation in the CNS [67–70]. The application of these advanced drug delivery systems may further optimize the therapeutic efficacy of the compound and support its future development for neurological and psychiatric disorders [71].

Regarding in vivo safety assessment, the absence of significant alterations in physiological parameters at the dose of 50 mg/kg indicates that, at this dose, the selected compounds **2** and **4** do not induce evident systemic toxicity. The maintenance of body weight, food intake, and water consumption suggests preservation of metabolic homeostasis and absence of acute discomfort or gastrointestinal impairment. Furthermore, the absence of alterations in locomotor and exploratory activity reinforces the notion that the compounds did not produce detectable neurobehavioral impairment under these conditions. Collectively, these findings corroborate a favorable acute safety profile at the lower dose and suggest an adequate tolerability margin for initial pharmacological evaluation.

Although ADMET predictions were generally favorable regarding compound safety, in vivo results in female mice indicated signs of toxicity with dose escalation for one of the selected compounds. The findings observed at 300 mg/kg highlight acute lethality induced by compound **2** (a smaller molecule compared to compound** 4**, which is bulkier) shortly after administration. Conversely, the absence of detectable physiological or behavioral alterations following administration of compound **4** at the same dose indicates a markedly distinct toxicological profile. Differences in toxicity profiles among molecules may be associated with structural features that influence their pharmacokinetic behavior, bioavailability, metabolic fate, and intrinsic chemical reactivity [72].

According to supplementary predictive analyses, both selected compounds **2** (aliphatic) and **4** (aromatic) exhibit high predicted probabilities of HLM instability, suggesting rapid hepatic metabolism and limited metabolic stability, with compound **4** showing the highest instability score. In contrast to the predicted LD_50_ values, compound **2** displayed higher lethality in the experimental assays and lower predicted lipophilicity compared to compound **4**. These differences may be associated with increased intrinsic chemical reactivity and/or broader systemic distribution of compound **2**, partially driven by its distinct LogP, LogD, and higher basic pKa values. These properties could favor greater dissolution in biological fluids, faster absorption, higher initial systemic exposure, and increased acute toxicity shortly after administration. Additionally, reduced lipophilicity of **2** may decrease plasma protein binding, increasing the free fraction available for distribution to target tissues. In contrast, the more lipophilic compound **4** could exhibit greater membrane partitioning, reducing its availability in aqueous biological compartments and limiting nonspecific interactions, which may contribute to its lower observed acute animal toxicity. Therefore, compound **2** is more acutely toxic possibly to its reactivity and wide distribution, while the less toxic compound **4** requires future chronic studies to investigate potential bioaccumulation.

Biochemical analyses demonstrated that AST activity remained unchanged at both tested doses, whereas ALT activity was significantly reduced at 300 mg/kg. The absence of alterations in AST, together with the absence of alterations in ALT at 50 mg/kg, suggests that the compounds did not induce hepatocellular damage under the evaluated experimental conditions. The significant reduction in ALT activity at the highest dose does not suggest classical hepatotoxicity but may instead reflect a modulatory effect on hepatic metabolism [73, 74]. As ALT is a key enzyme involved in amino acid metabolism and gluconeogenesis [75], a decrease in its activity may indicate metabolic adaptation or modulation of transamination processes. Finally, the absence of concomitant changes in AST reinforces the interpretation that liver integrity was preserved, and these results corroborate the hepatic safety of the compounds in the tested range.

Respectively, biochemical analysis revealed that serum levels of total proteins and urea did not show significant differences between treated groups and the control group. The maintenance of total protein concentrations suggests absence of impairment in hepatic function; similarly, the stability of urea levels indicates that there was no significant compromise of renal function. These findings corroborate the preliminary safety profile of the tested compounds, indicating no relevant systemic impact on classical biochemical parameters of hepatic and renal function.

The apparent discrepancy observed in the 300 mg/kg group, characterized by increased lipid peroxidation concomitant with reduced ALT levels, can be explained by the fact that these parameters reflect different physiological processes reflected by these biomarkers. While ALT is a marker of hepatocellular membrane damage and leakage of enzymes into the circulation, lipid peroxidation primarily indicates oxidative damage to membrane lipids. Thus, an increase in lipid peroxidation does not necessarily translate into hepatocellular injury extensive enough to raise serum ALT levels [76]. It is possible that, at this dose, the treatment induced a moderate oxidative challenge, as evidenced by the increase in lipid peroxidation, without corresponding impairment of hepatocellular integrity and limiting enzyme release. This interpretation is further supported by the absence of alterations in complementary plasma biochemical parameters (plasma AST and total proteins). In addition, adaptive cellular responses, including the activation of antioxidants and cytoprotective pathways, may have contributed to the maintenance of liver function, despite the increase in OS markers [77].

The superior performan**ce **of compound **4** is likely related to the meta positioning of the ester and selenocyanate groups on the aromatic ring, which minimizes unfavorable resonance and steric effects and helps preserve an optimal electronic environment at the selenium center, probably enhancing antioxidant activity, enzyme inhibition, and safety profile. Taken together, these findings support a multimodal antioxidant mechanism involving free radical scavenging, electron donation, and attenuation of OS in biological systems. Such redox-modulating properties are particularly relevant in the context of neurodegenerative diseases, where persistent OS contributes to neuronal dysfunction, mitochondrial impairment, and progressive cell loss.

Furthermore, compound **4** emerged as the most promising candidate among the compounds evaluated due to its consistent antioxidant activity across different assays, combined with a favorable safety profile, as no significant toxicity was observed at the highest dose tested (300 mg/kg). These findings suggest that compound **4** possesses an advantageous balance between biological activity and tolerability. To further validate its therapeutic potential, future studies should investigate its efficacy in models of diseases associated with OS, as well as its chronic toxicity profile. Furthermore, pharmacokinetic and pharmacodynamic (PK/PD) studies will be important to characterize its absorption, distribution, metabolism, and elimination, providing a more comprehensive assessment of its suitability for preclinical development.

## Conclusion

This study described the synthesis and the in silico, in vitro, and in vivo bioactivities of five organic selenocyanate derivatives. All compounds exhibited antioxidant effects, with compounds **1** , **4**, and **5** showing the most prominent activity in a battery of in vitro tests. The antioxidant mechanisms identified, which varied depending on the compound, appear to be mediated primarily by ET and GST-mimetic activity. Additionally, all five compounds inhibited cerebral MAO A and B activities, suggesting potential modulation of monoamine levels and redox balance. All derivatives also inhibited cerebral AChE activity, with compounds **2** and **4** being the most effective AChE inhibitors. Molecular docking studies revealed probable interactions of the compounds with these neurochemical enzymes. Predicted ADME/T profiles indicated relative safety for all compounds; however, in vivo toxicity studies raised particular concerns regarding compound **2**. Some predicted pharmacokinetic limitations were also reported. These findings suggest that compound 4 possesses an advantageous balance between biological activity and tolerability. Overall, this integrated evaluation highlights selenocyanates derivatives as promising multitarget, redox-active neuroprotective candidates.

## Supplementary Information

Below is the link to the electronic supplementary material.

Supplementary Material 1 (DOCX 7.74 MB)

## Data Availability

The data are available from the corresponding author, CFB, upon reasonable request.

## Declarations

### Generative Artificial Intelligence (AI)

AI-assisted tools (ChatGPT-5 and Gemini) were used exclusively for grammar checking and spelling, while Consensus was used for scientific literature retrieval and screening.
