# Supplementary material for: An Integrated in Silico, in Vitro, and in Vivo Assessment of the Antioxidant and Neuroprotective Profile of Selenocyanate Compounds
Source: Biol Trace Elem Res. 2026 Jul 31;204(10):8107–29. doi: 10.1007/s12011-026-05247-7 (PMC13616007; doi:10.1007/s12011-026-05247-7)
Supplement: Supplementary file 1 [file 12011_2026_5247_MOESM1_ESM.docx]

**SUPPLEMENTARY MATERIAL**

**An integrated in silico, in vitro, and in vivo assessment of the antioxidant and neuroprotective profile of selenocyanate compounds**

Maria Eduarda Ribeiro da Rocha^a^, Bruna Rafaela Pizzi^a^, Pâmella da Costa^a^, Tácia Katiane Hall^a^, Mariana Parron Paim^a^, Pâmella Cordeiro^b^, Victor Menezes^b^, Vanessa Nascimento^b^, César Augusto Brüning^a^, Paloma Taborda Birmann^ac2^ and Cristiani Folharini Bortolatto^a1^

^a^Laboratory of Biochemistry and Molecular Neuropharmacology (LABIONEM), Graduate Program in Biochemistry and Bioprospecting (PPGBBio), Chemical, Pharmaceutical, and Food Sciences Center (CCQFA), Federal University of Pelotas (UFPel), 96010-900 (RS, Brazil).

^b^SupraSelen Laboratory, Chemistry Institute, Department of Organic Chemistry, Federal Fluminense University (UFF), Niterói, Rio de Janeiro, Brazil.

^c^Research Group in Biochemistry and Toxicology in Eukaryotes, Postgraduate Program in Biochemistry, Federal University of Pampa (UNIPAMPA), Bagé (RS, Brazil).

CONTENT SUMMARY

[EXPERIMENTAL - CHEMICAL SYNTHESIS	2](#_Toc233152051)

[FIGURES....................	4](#_Toc233152052)

[1.	1H NMR and 13C-APT NMR spectra	4](#_Toc233152053)

[2.	Molecular Docking	9](#_Toc233152054)

[TABLE…………………………………………………………………………………………………………………	18](#_Toc233152055)

[Predicted ADME/T physicochemical properties of the selected selenocyanates	18](#_Toc233152056)

# EXPERIMENTAL - CHEMICAL SYNTHESIS

**General information.** All solvents and reagents used in the synthesis, purification and characterization were purchased from commercial sources Sigma-Aldrich, Merck (Darmstadt, Germany) and Synth (Sao Paulo, Brazil) and used without prior purification. For the procedure of purification of the compounds by using column chromatography, the material applied was a glass column, as stationary phase silica gel was used with a 0.063 - 0.2 mesh by Merck (Darmstadt, Germany) and, as eluent, a suitable solvent or solvent mixture. The fractions and compounds obtained were analyzed by thin layer chromatography (TLC), using aluminum plates coated with silica gel 60 GF 254, provided by Merck (Darmstadt, Germany), 0.25 mm thick and with particles between 5 and 40 µm in diameter. The substances separated on the chromatographic plates were visualized by using several development methods: in an iodine chamber, in an ultraviolet light chamber, or with a vanillin reagent followed by heating. APPI-Q-TOFMS measurements were taken on a mass spectrometer equipped with an automatic syringe pump for sample injection. The ^1^H and ^13^C nuclear magnetic resonance spectra were obtained by using the Varian AS-400 spectrometer, operating at 500 MHz and 300 MHz for ^1^H and 125 MHz for ^13^C. Chemical shifts are reported in ppm, referenced to the solvent peak of CDCl_3_ or TMS. Data are reported as follows: chemical shift (δ), multiplicity, coupling constant (*J*) in hertz and integrated intensity. Standard abbreviations were used to denote the multiplicity of a particular signal (s = singlet; d = doublet; t = triplet; q = quartet; m = multiplet).

**General procedure for the synthesis of compounds 1 and 2.** In a round bottom flask equipped with a reflux condenser, the respective bromide **EST- 1c** or **EST-2c** was added in 0.76ml of acetonitrile and allowed to stir at room temperature. After that, 1.5 equivalents (equiv.) of potassium selenocyanate was added to the system. The reaction was stirred overnight at 80°C. At the end of the time, the flask was cooled to room temperature, and the crude product was extracted with ethyl acetate and water. The **1** or **2** products were purified with column chromatography using Hexane/Ethyl acetate mixture.

Ethyl 2-selenocyanatoacetate (**1**): 90% yield; Orange oil; IR ʋ_max_ (cm^-1^; KBr): 2982, 2151, 1724. ^1^H NMR (500 MHz, CDCl_3_) δ 4.26 (q, *J* = 5 Hz, 2H), 3.84 (s, 2H), 1.32 (t, *J* = 5 and 10Hz, 3H). ^13^C NMR (126 MHz, DMSO-d_6_) δ 168.91, 103.90, 61.95, 28.52, 14.36.^16^

Ethyl 3-selenocyanatopropanoate (**2**): 70% yield; Light yellow oil; IR ʋ_max_ (cm^-1^; KBr): 2926, 2150, 1727. ^1^H NMR (500 MHz, CDCl_3_) δ 4.17 (q, J = 5 Hz, 2H), 3.23 (t, *J* = 5 and 10Hz, 2H), 2.95 (t, *J* = 5 and 10 Hz, 2H), 1.26 (t, *J* = 5 Hz, 1H). ^13^C NMR (126 MHz, DMSO-d_6_) δ 170.80, 103.76, 60.32, 35.01, 23.72, 13.82.^16^

**General procedure for the synthesis of compounds 3**-**5.** In a round-bottom flask, the corresponding ester **3** was combined with 0.48 mL of 50% aqueous HBF_4_. The mixture was stirred in an ice-salt bath (0-5°C), and a 14% aqueous NaNO_2_ solution (0.6 mL) was added dropwise. The system was stirred for 30 minutes, and the flask was kept covered with aluminum foil during this step. The pH of the reaction mixture was then adjusted to 6 using a saturated AcONa solution. Subsequently, potassium selenocyanate was added slowly, and the mixture was stirred at room temperature for 5-6 hours. After completion, distilled water (30 mL) was added, followed by extraction with DCM (3 × 15 mL) and washing with distilled water. The organic layer was dried over Na_2_SO_4_, filtered, and concentrated under reduced pressure. The crude products were purified by column chromatography using a hexane/DCM gradient, affording the aromatic selenocyanates in 30-90% yield.

Ethyl 2-selenocyanatobenzoate (**3**): 90% yield; Orange oil; IR ʋ_max_ (cm^-1^; KBr): 2982, 2151, 1724. ^1^H NMR (500 MHz, CDCl_3_) δ 8.07 (d, J = 8.0 Hz, 1H), 8.00 (d, J = 8.0 Hz, 1H), 7.54 (t, J = 8.0 Hz, 1H), 7.37 (t, J = 8.0 Hz, 1H), 4.38 (q, J = 7.1 Hz, 2H), 1.36 (t, J = 7.1 Hz, 3H). ^13^C (125 MHz, DMSO-*d*_6_) δ (ppm) = 164,4; 145,3; 137,3; 133,2; 131,0; 129,7; 128,8; 126,8; 60,7; 13,6.^17^

Ethyl 3-selenocyanatobenzoate (**4**): 30% yield; Brown oil; IR ʋ_max_ (cm^-1^; KBr): 2987, 2149, 1707. ¹H NMR (500 MHz, CDCl_3_) δ 8.29 (s, 1H), 8.10 (d, J = 5 Hz, 1H), 7.84 (d, J = 5 Hz, 1H), 7.50 (t, J = 5 Hz, 1H), 4.40 (q, J = 5 Hz, 2H), 1.41 (t, J = 5 Hz, 3H). RMN ^13^C (125 MHz, DMSO-d^6^) δ (ppm) = 164,4; 145,3; 137,3; 133,2; 131,0; 129,7; 128,8; 126,8; 60,7; 13,6.^17^

Ethyl 4-selenocyanatobenzoate (**5**): 65% yield; Yellow oil; IR ʋ_max_ (cm^-1^; KBr): 2987, 2150, 1724. ¹H NMR (500 MHz, CDCl_3_) δ 7.94 (d, J = 8.1 Hz, 2H), 7.50 (d, J = 8.1 Hz, 2H), 4.37 (q, J = 7.1 Hz, 2H), 1.39 (t, J = 7.1 Hz, 3H). ^13^C NMR (125 MHz, DMSO-*d*_6_) δ 165.20, 144.80, 133.82, 132.02, 130.13, 129.49, 60.96, 14.12.^17^

# FIGURES

## 1. 1H NMR and 13C-APT NMR spectra

Fig. S1. ^1^H NMR (500 MHz, CDCl_3_) spectrum of compound **1**.

Fig. S2. ^13^C-APT NMR (125 MHz, CDCl_3_ ) spectrum of compound 1.

Fig. S3. ^1^H NMR (500 MHz, CDCl_3_) spectrum of compound **2**.

Fig. S4. ^13^C-APT NMR (125 MHz, CDCl_3_ ) spectrum of compound **2**.

Fig. S5. ^1^H NMR (500 MHz, CDCl_3_) spectrum of compound **3**.

Fig. S6. ^13^C-APT NMR (125 MHz, CDCl_3_ ) spectrum of compound **3**.

Fig. S7. ^1^H NMR (500 MHz, CDCl_3_) spectrum of compound **4**.

Fig. S8. ^13^C-APT NMR (125 MHz, CDCl_3_) spectrum of compound **4**.

Fig.S9. ^1^H NMR (500 MHz, CDCl_3_) spectrum of compound **5**.

Fig. S10. ^13^C-APT NMR (125 MHz, CDCl_3_) spectrum of compound **5**.

## 2. Molecular Docking

Fig. S11. 3D binding pose of compound **1** in acetylcholinesterase.

Fig. S12. 3D binding pose of compound **2** in acetylcholinesterase.

Fig. S13. 3D binding pose of compound **3** in acetylcholinesterase.

Fig. S14. 3D binding pose of compound **4** in acetylcholinesterase.

Fig. S15. 3D binding pose of compound **5** in acetylcholinesterase.

Fig. S16. 2D binding pose of positive control rivastigmine in acetylcholinesterase.

Fig. S17. 3D binding pose of compound **1** in monoamine oxidase A.

Fig. S18. 3D binding pose of compound **2** in monoamine oxidase A.

Fig. S19. 3D binding pose of compound **3** in monoamine oxidase A.

Fig. S20. 3D binding pose of compound **4** in monoamine oxidase A.

Fig. S21. 3D binding pose of compound **5** in monoamine oxidase A.

Fig. S22. 2D binding pose of positive control clorgyline in monoamine oxidase A.

Fig. S23. 3D binding pose of compound **1** in monoamine oxidase B.

Fig. S24. 3D binding pose of compound **2** in monoamine oxidase B.

Fig. S25. 3D binding pose of compound **3** in monoamine oxidase B.

Fig. S26. 3D binding pose of compound **4** in monoamine oxidase B.

Fig. S27. 3D binding pose of compound **5** in monoamine oxidase B.

Fig. S28. 2D binding pose of positive control pargyline in monoamine oxidase B.

# TABLE

## Predicted ADME/T physicochemical properties of the selected selenocyanates

**Table S1.** Predicted ADME/T physicochemical properties of the selected selenocyanates

|  | | Compounds | | | |  |
| --- | --- | --- | --- | --- | --- | --- |
| Property | **2** | | **4** | **Comment** |  |  |
| HLM Stability | 0.983 | | 0.999 | 0: stable+ (HLM>30min); 1: unstable (HLM≤30min) | | |
| LogP | 0.836 | | 2.422 | The logarithm of the n-octanol/water distribution coefficients at pH=7.4 | | |
| LogD | 0.647 | | 2.356 | The logarithm of the n-octanol/water distribution coefficient | | |
| pKa (acid) | 8.385 | | 8.368 | Acid-base dissociation constant | | |
| pKa (basic) | 4.825 | | 2.822 | Acid-base dissociation constant | | |
